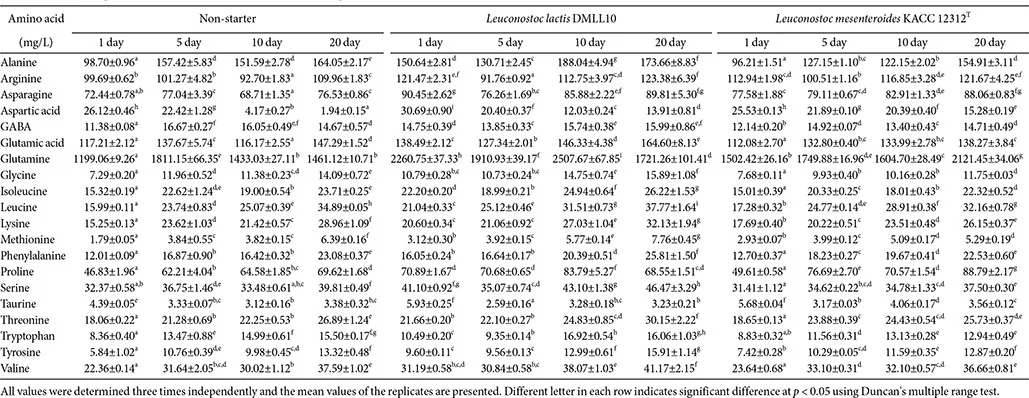# Comparative Effects of *Leuconostoc lactis* and *Leuconostoc mesenteroides* Starters on Kimchi Fermentation Dynamics and Metabolite Profiles

**DOI:** 10.4014/jmb.2609.09002

**Published:** 2026-09-23

**Authors:** Yura Moon, Sojeong Heo, Gawon Lee, Junghyun Park, Seung-Eun Oh, Sumin Lee, Minkyeong Kim, Do-Won Jeong

**Affiliations:** 1 Department of Food and Nutrition, Dongduk Women’s University, Seoul 02748, Republic of Korea; 2 Dairy Products Team, Product Development Center, R&BD Division, hy Co., Ltd., Yongin-si, Gyeonggi-do, 17086, Republic of Korea

**Keywords:** Starter, Kimchi fermentation, Organic acids, Amino acids, Volatile compounds

## Abstract

This study evaluated the effects of two *Leuconostoc* starter—*Leuconostoc* (*Leu.*) lactis DMLL10 and *Leu. mesenteroides* KACC 12312^T^—on kimchi fermentation characteristics, microbial dynamics, and metabolite production. Kimchi samples were fermented at 4°C for 20 days, and changes in pH, titratable acidity, viable cell counts, organic acids, amino acids, and volatile compounds were analyzed using GC–MS, HPLC, and multivariate statistical methods. Both starters successfully initiated fermentation, but clear differences in metabolic patterns were observed. *Leu. lactis* DMLL10-inoculated kimchi showed a lower relative abundance of *Latilactobacillus sakei*, a homofermentative LAB associated with excessive acidification, than the control. Compared with *Leu. mesenteroides*, *Leu. lactis* DMLL10-inoculated kimchi showed a relatively lower final titratable acidity and generated a broader range of organic acids, amino acids, and volatile compounds. In particular, it produced higher levels of glutamic acid, contributing to umami taste, as well as unique volatiles such as octanoic acid and specific aldehydes, enhancing flavor complexity. In contrast, *Leu. mesenteroides* KACC 12312^T^ showed faster acidification and a comparatively narrower metabolite profile. Multivariate and correlation analyses (PCA, OPLS-DA, and network mapping) revealed distinct fermentation patterns between the two tested starter strains. These findings support *Leu. lactis* DMLL10 as a promising alternative starter candidate for diversifying kimchi fermentation profiles, although further studies using multiple strains are required to determine whether the observed differences are species-specific or strain-dependent differences.

## Introduction

*Baechu-kimchi* made from *baechu* (kimchi cabbage; *Brassica rapa*) is the most commonly consumed fermented vegetable food in Korea [1]. The microorganisms involved in the fermentation of *baechu-kimchi* are mainly lactic acid bacteria (LAB). It is well known that *Leuconostoc* species dominate in the early stages of fermentation, which is the period most preferred by consumers [2-5]. *Leuconostoc* species is a heterofermentative LAB that produces lactic acid, CO_2_, from glucose via heterolactic fermentation. In addition, they can contribute to the formation of aroma compounds, including diacetyl, through metabolic pathways such as citrate metabolism. As a result, unlike raw *baechu*, kimchi develops sourness, distinctive flavors, and umami characteristics during fermentation [6, 7]. Following *Leuconostoc* species, members of the genus *Lactobacillus* and related LAB often increase during later stages of kimchi fermentation [8, 9]. Some late-stage LAB, including several *Lactobacillus* species, are known to be homofermentative bacteria that convert one molecule of glucose into two molecules of lactic acid, which may contribute to increased sourness and reduced consumer preference during kimchi fermentation [10-12]. Therefore, much effort has been made in commercial kimchi production to prolong the early fermentation stage dominated by *Leuconostoc* [13-15].

Currently, the starter cultures used in commercial kimchi production in Korea include members of the genus *Leuconostoc*, particularly *Leuconostoc* (*Leu.*) *mesenteroides*. *Leu. mesenteroides* is the most frequently detected and isolated species in fermented kimchi [2-5, 16]. However, other species such as *Leu. carnosum*, *Leu. citreum*, and *Leu. lactis* are also occasionally identified [3, 4, 17]. Although a few studies have proposed novel *Leuconostoc* species as potential starter candidates for kimchi fermentation, none have yet been applied in commercial production. Moreover, there are very few reports that have directly compared fermentation characteristics among different *Leuconostoc* species when applied to kimchi fermentation, despite the recognized diversity within the genus.

In our previous study, we investigated whether the addition of seafood ingredients could alter the microbial community structure in *baechu*-kimchi [3, 4]. From kimchi samples, we isolated a total of 816 LAB isolates. Among them, 534 isolates belonged to the genus *Leuconostoc*, with *Leu. mesenteroides* being the most dominant (469 isolates, 57.9%), followed by *Leu. citreum* (49 isolates, 6.0%), *Leu. carnosum* (9 isolates, 1.1%), and *Leu. lactis* (4 isolates, 0.5%). Among these, *Leuconostoc lactis* was subjected to a safety evaluation and fermentation suitability test, leading to the selection of strain DMLL10 as a potential starter candidate [18]. Strain DMLL10 was sensitive to eight antibiotics, showed protease activity at 3% NaCl (a typical salt concentration in kimchi), and exhibited antimicrobial activity against seven spoilage or foodborne pathogenic bacteria [18]. Furthermore, genetic toxicity assessment confirmed that DMLL10 had no genotoxic effects [19]. These results indicate that strain DMLL10 is a safe starter candidate that may contribute to flavor and aroma development through enzymatic activity and promote safe fermented food production by inhibiting foodborne pathogens.

However, the direct functionality of this strain as a starter in kimchi fermentation has not yet been experimentally validated, and—more importantly—its fermentation characteristics have not been compared with those of the commonly used starter, *Leu. mesenteroides*. Such comparison is essential to determine whether strain DMLL10 can serve as a viable alternative or complementary starter in commercial contexts. Therefore, this study aimed to investigate, for the first time, the effects of *Leu. lactis* DMLL10 as a starter on the microbial community, organic acids, amino acids, and volatile compounds during kimchi fermentation. Additionally, *Leu. mesenteroides* was used as a control to comparatively analyze the fermentation products. The findings of this study are expected to provide a scientific basis for strategic and evidence-based starter selection in fermented food production.

## Materials and Methods

### Bacterial Strains and Culture Conditions

*Leu. lactis* DMLL10 from kimchi was used as a starter strain for kimchi fermentation. And the type strain *Leu. mesenteroides* KACC 12312^T^ (=ATCC 8293^T^) was used as a control strain. *Leuconostoc* species were cultured in MRS broth (MB Cell, Republic of Korea) at 30°C for 16 h. The culture was then inoculated into fresh MRS medium at 1% (v/v) and incubated at 30°C. When the culture reached an optical density of 0.7 at 600 nm, the culture was used as a starter for kimchi.

### Kimchi Preparation and Fermentation

Kimchi was prepared following the method described in our previous study [20]. Briefly, salted cabbage (2% NaCl, 100 g) was cut into 1 × 1 cm² pieces and mixed with green onion (3.1 g), red pepper powder (3.1 g), garlic (1.5 g), and ginger (0.4 g). For each experimental group, three independent biological replicates were prepared as independently manufactured kimchi batches. Each batch consisted of 100 g of salted cabbage and separately weighed ingredients, which were individually mixed and inoculated with the corresponding starter culture of left uninoculated for the control group. Kimchi without starter inoculation was used as the control (non-starter sample). For the starter-inoculated groups, cultures of *Leu. lactis* DMLL10 and *Leu. mesenteroides* KACC 12312^T^ were added to the kimchi at a final concentration of approximately 10^6^ CFU/g. After preparation, kimchi samples were fermented at 4°C, and aliquots were collected on days 1, 5, 10, 15, and 20. All samples were stored at -80°C until analysis.

### pH, Total Titratable Acidity and Viable Cell Counts Analysis

To measure pH, titratable acidity, and viable LAB counts during kimchi fermentation, kimchi samples were homogenized using a mixer (BL133AKR, Tefal, France). Ten grams of the homogenized sample were taken and diluted tenfold with sterile distilled water. The diluted mixture was then filtered through filter paper, and the filtrate was used for subsequent analyses. The pH of the filtrate was determined using a pH meter (MP220, Mettler-Toledo, USA). Titratable acidity was measured according to the standard analytical method (6.12.2.3) described in the Korean Food Code issued by the Ministry of Food and Drug Safety, using phenolphthalein as an indicator and titration with 0.1 N NaOH. For viable cell counts, the filtrate was serially diluted with sterile water according to the decimal dilution method and spread-plated onto MRS agar (MB Cell). The plates were incubated at 30°C for 48 h. The growth of LAB in kimchi was expressed as log CFU/g. All analyses were performed in triplicate.

### 16S rRNA Gene Amplicon Sequencing for Kimchi Samples

To assess the impact of starter culture addition on the bacterial community structure, 16S rRNA gene amplicon sequencing analysis was conducted. Genomic DNA was extracted from kimchi sample filtrates using the Maxwell RSC PureFood GMO and Authentication Kit (Promega, USA), in accordance with the manufacturer’s instruction. The V3–V4 hypervariable regions of the bacterial 16S rRNA genes were amplified using fusion primer set 341F (5’-AAT GAT ACG GCG ACC ACC GAG ATC TAC AC-X XXX XXX X-TC GTC GGC AGC GTC-AGA TGT GTA TAA GAG ACA G-CC TAC GGG NGG CWG CAG-3’; underlining sequence indicates the target region primer) and 805R (5’- CAA GCA GAA GAC GGC ATA CGA GAT-XXX XXX XX-G TCT CGT GGG CTC GG-A GAT GTG TAT AAG AGA CAG-GAC TAC HVG GGT ATC TAA TCC-3’). The fusion primers are constructed in the following order which is P5 (P7) graft binding, i5 (i7) index, Nextera consensus, sequencing adaptor, and target region sequence. The PCR conditions included an initial denaturation at 95°C for 3 min; 25 cycles of denaturation at 95°C for 30 seconds, annealing at 55°C for 30 sec, and extension at 72°C for 30 sec; followed by a final extension at 72°C for 5 min. The PCR products were confirmed by electrophoresis on a 1% agarose gel and visualized using a Gel Doc system (BioRad, USA). The amplified products were purified using CleanPCR (CleanNA, Netherlands Netherlands), pooled at equal concentrations, and removed short fragments (non-target products) with CleanPCR. Product quality and size were evaluated using a Bioanalyzer 2100 (Agilent, USA) with a DNA 7500 chip. Mixed amplicons were pooled and 16S rRNA gene amplicon sequencing was performed by CJ Bioscience, Inc. (Republic of Korea), with the Illumina MiSeq Sequencing system (Illumina, USA), following the manufacturer’s protocols.

Quality control began with Trimmomatic (ver. 0.32), which removed low-quality reads (<Q25) [21]. After passing QC, paired-end sequences were merged using the fastq_mergepairs command in VSEARCH (ver. 2.13.4) with default parameters [22]. Taxonomic assignments were made using the EzBioCloud 16S rRNA database [23]. Chimeric reads were filtered on reads with <97% similarity by reference based chimeric detection using UCHIME algorithm and the non-chimeric 16S rRNA database from EzBioCloud [24]. Reads not identified at the species level in the EzBioCloud database (<97% similarity) were compiled and subjected to de-novo clustering with the cluster_fast command to generate additional OTUs. Finally, OTUs represented by a single read (singletons) were excluded from further analysis. The alpha diversity indices analysis which includes Chao1, Shannon was conducted by in-house programs of CJ Bioscience. All analytics mentioned above were performed in EzBioCloud 16S-based MTP, which is a CJ Bioscience’s bioinformatics cloud platform.

### Analysis of Organic Acids

Ground kimchi samples (5 g) were mixed with 20 mL of sterilized water. The mixture was sonicated for 10 min, and then filtered through a 0.22 μm Millex-GV syringe-driven filter unit (Merck Millipore, Germany). The filtrate was analyzed by a custom service provided by the National Instrumentation Center for Environmental Management in Korea (NICEM; http://nicem.snu.ac.kr/). The analyses were performed using the Dionex Ultimate 3000 HPLC system (Thermo Fisher Scientific, USA). Chromatographic separation was conducted with an Aminex 87H column (300 mm × 10 mm; Bio-Rad). Isocratic elution was carried out using 0.01N H_2_SO_4_ as the mobile phase. Injection volumes were 10 μL, and the column temperature and flow rate were 40°C and 0.5 mL/min, respectively. The HPLC system was equipped with a refractive index (RI) detector (RefractoMAX520, ERC Inc., Japan) and a UV detector operated at 210 nm. Organic acid concentrations determined by HPLC were expressed as mg/L of the aqueous extract. The organic acid contents of three samples prepared in the same conditions were analyzed in triplicate.

### Analysis of Free Amino Acids

The same filtrate that was used for the analysis of organic acids was analyzed at NICEM. The analyses were performed using a Dionex Ultimate 3000 HPLC system. Chromatographic separation was conducted with an Inno C18 column (4.6 mm × 150 mm, 5 µm; Youngjin Biochrom, Republic of Korea). Gradient elution was carried out with 40 mM sodium phosphate (solvent A; pH 7) and methanol/acetonitrile/water (solvent B; 45:45:10 v/v). The following binary mobile phase linear gradients were used: 95 % A at 0 min, 95% A at 3 min, 45% A at 24 min, 10% A at 25 min, 10% A at 31 min, 95% A at 34 min, and 95% A at 35 min. Injection volumes were 0.5 μL, and the column temperature and flow rate were 40°C and 1.5 mL/min, respectively. The detection was performed using a Agilent 1260 infinity FL detector (Agilent). OPA reagent (Agilent) and FMOC solution (Agilent) was used as derivatizing agents, according to the manufacturer’s instructions. The fluorescence detection was set at an excitation wavelength of 340 nm and an emission wavelength of 450 nm for the determination of the OPA reagent-derivatized amino acids, whereas an excitation wavelength of 266 nm and an emission wavelength of 305 nm were used for the determination of the FMOC reagent-derivatized amino acids. The concentrations of individual free amino acids were determined using five-point calibration curves of the amino acid standard (Agilent 5061-3330, Agilent 5062-2478). Amino acid concentrations determined by HPLC were expressed as mg/L of the aqueous extract. The free amino acid contents of three samples prepared in the same conditions were analyzed in triplicate.

### Analysis of Volatile Compounds

One gram of kimchi sample was mixed with 3.25 mL of sterilized water and 0.15 g of NaCl in a 15 mL vial with a tan polytetrafluoroethylene/silicone septum (tan PTFE/silicone septum; Supelco, USA). The sample prepared in this way was then kept at 60°C for 30 min. The volatile compounds were absorbed onto a 50/30 μm divinylbenzene/carboxen/polydimethylsiloxane fiber (DVB/CAR/PDMS fiber; Supelco) for 15 min, and then eluted at 220°C. The volatile compounds were transferred through the MS transfer line at 230°C (ISQ 7000^TM^ Single Quadrupole GC-MS system; Thermo Fisher Scientific). Separation was performed on a TG-WAXMS GC column (30 m length × 0.25 mm i.d. × 0.25 μm film thickness; Thermo Fisher Scientific). The oven program was held at 40°C for 3 min, raised at 2°C/min to 150°C and then held at 150°C for 10 min, and raised again at 4°C/min to 200°C and held at 200°C for 10 min; the carrier gas (He) flow rate was 1.0 mL/min, ionization energy was 70 eV, and the mass scan range was 50−550 *m/z*. Chromeleon Chromatography Data System Software (ver. 7.2.10) was used to pre-process raw GC-MS data (Dionex, USA).

The internal standard was methyl cinnamate (99%) solution at 1.5 μL of 1,000 ppm. The quantitative data were acquired by comparing the peak area (min × counts) of the experimental value obtained by including 1-fold, 2-fold, and 3-fold internal standards excluding kimchi samples, represented by a linear equation. The analysis of volatile compounds was conducted in triplicate for each sample.

### Pearson Correlation Analysis

Pearson correlation analysis was performed to examine the relationships between the three kimchi samples and their metabolites. The Pearson correlation coefficients were analyzed using PAST software (ver. 4.10; University of Oslo, Norway), and values with *p* < 0.05 were considered statistically significant. In addition, Cytoscape software (ver. 3.10.3; Cytoscape Consortium, USA) was used to visualize the Pearson correlation coefficients [25].

### Statistical Analysis

At each fermentation time point, Duncan's multiple range test following a one-way analysis of variance (ANOVA) was used to evaluate significant differences among the treatment groups in the mean values of physicochemical properties and metabolites. Values with *p* < 0.05 were considered to be statistically significant using SPSS software (ver. 29.0; IBM, Armonk, NY, USA). To visualize metabolic differences among the three kimchi samples, principal component analysis (PCA) was performed using the concentrations of all detected metabolites after mean-centering and unit variance (UV) scaling in SPSS software. In addition, orthogonal partial least squares discriminant analysis (OPLS-DA) was conducted using SIMCA software (ver. 18.1.0.507; Sartorius Stedim Data Analytics AB, Umeå, Sweden). Prior to OPLS-DA, the data were mean-centered and UV-scaled. Model performance was evaluated using the cumulative explained variance (R²X and R²Y) and predictive ability (Q²) obtained by seven-fold cross-validation. To assess model robustness and avoid overfitting, permutation testing (200 permutations) was performed.

## Results and Discussion

### Changes in pH, Acidity, and Viable Cell Counts

In general, the pH of kimchi decreases while acidity and viable cell counts increase as fermentation progresses [3, 8, 9, 12]. To assess whether fermentation proceeded properly and to identify any differences among starter strains, these parameters were monitored (Fig. 1A and Table S1).

In kimchi inoculated with *Leu. lactis* DMLL10, the pH decreased from 5.25 ± 0.04 on day 1 to 4.01 ± 0.01 on day 20 of fermentation. Similarly, the pH of non-starter kimchi and kimchi inoculated with *Leu. mesenteroides* KACC 12312^T^ decreased from 5.22 ± 0.02 and 5.21 ± 0.03 on day 1 to 4.07 ± 0.01 and 3.85 ± 0.01 on day 20, respectively. The overall pattern of pH change was similar among groups, with a sharp decrease observed on day 5. Among the samples, kimchi inoculated with *Leu. mesenteroides* KACC 12312^T^ showed the lowest pH at day 20 (Fig. 1A and Table S1).

The decrease in pH corresponded to an increase in acidity. In kimchi inoculated with *Leu. lactis* DMLL10, the acidity increased from 0.37 ± 0.02%on day 1 to 0.88 ± 0.04% on day 20. Non-starter kimchi and *Leu. mesenteroides* KACC 12312^T^-inoculated kimchi showed initial acidities of 0.32 ± 0.03% and 0.35 ± 0.00%, respectively, and increased to 0.82 ± 0.04% and 0.94 ± 0.05% on day 20. Although acidity did not increase as sharply as pH decreased, especially around day 5, all samples showed a gradual upward trend. This general increase in acidity, together with the decrease in pH, indicates that fermentation proceeded in all kimchi samples. Among the samples, *Leu. mesenteroides* KACC 12312^T^-inoculated kimchi showed the lowest pH and highest acidity on day 20, whereas *Leu. lactis* DMLL10-inoculated kimchi showed a final titratable acidity higher than that of non-starter kimchi but lower than that of *Leu. mesenteroides* KACC 12312^T^-inoculated kimchi under the present experimental conditions. These differences suggest that the extent of acidification and the organic acid production profiles may vary depending on the inoculated starter strain (Fig. 1A and Table S1). However, the observed acidity differences should be interpreted descriptively rather than as evidence of controlled fermentation or prevention of over-acidification. Further studies using predefined quality criteria and sensory evaluation are needed to determine whether these differences have practical significance for kimchi quality.

Viable cell counts in *Leu. lactis* DMLL10-inoculated kimchi increased from 6.01 ± 0.05 log CFU/g on day 1 to 7.62 ± 0.01 log CFU/g on day 5, then decreased to 6.46 ± 0.07 log CFU/g by day 20. In *Leu. mesenteroides* KACC 12312^T^-inoculated kimchi, the viable cell counts were higher than that of *Leu. lactis* DMLL10-inoculated kimchi from day 5 onward, reaching 8.28 ± 0.04 log CFU/g on day 5 and remaining the highest among the three groups at 6.88 ± 0.26 log CFU/g on day 20. In contrast, the non-starter kimchi showed the lowest viable counts overall, increasing from 3.28 ± 0.07 log CFU/g on day 1 to 7.33 ± 0.11 log CFU/g on day 10, and then decreasing to 6.15 ± 0.05 log CFU/g on day 20 (Fig. 1A and Table S1).

These results demonstrate that the viable bacterial populations were higher in kimchi inoculated with starter cultures and that the total viable counts differed depending on the inoculated strain. Moreover, the marked increase in viable cells observed on day 5, coinciding with the sharp decline in pH, suggests that fermentation at 4°C proceeded actively up to approximately day 5.

### Verification of Starter Dominance during Kimchi Fermentation

To verify the dominance of the inoculated starter strains during kimchi fermentation, the microbial community was analyzed by 16S rRNA gene amplicon sequencing (Fig. 1B). At the phylum level, Firmicutes and Proteobacteria were predominant. Other minor phyla accounted for 2.6–6.6% of the total microbiota on day 1 but decreased to 0.1–0.4% after day 5 of fermentation. In kimchi inoculated with *Leu. lactis* DMLL10, Firmicutes and Proteobacteria were present at 75.7% and 21.7%, respectively, on day 1; by day 5, Firmicutes increased to 87.1%, while Proteobacteria decreased to 12.8%, with Firmicutes continuing to increase as fermentation progressed. Kimchi inoculated with *Leu. mesenteroides* KACC 12312^T^ showed a similar trend, though Proteobacteria were slightly more abundant than in the *Leu. lactis* DMLL10-inoculated kimchi. In contrast, Proteobacteria in non-starter kimchi accounted for 41% on day 1 but gradually decreased to 6.4 % by day 20 (Fig. 1B).

At the genus level, the Firmicutes detected in *Leu. lactis* DMLL10-inoculated kimchi on day 1 were mainly *Leuconostoc* (55.4%), *Bacillus* (10.5%), and *Lactobacillus* (4.3%). By day 5, *Leuconostoc* increased to 64.8 %, followed by *Lactobacillus* (21.6%), *Bacillus* (0.3%), and *Weissella* (0.1%). On day 20, *Leuconostoc* (70.4%), *Lactobacillus* (24.3%), and *Weissella* (0.7%) were predominant. In *L. mesenteroides* KACC 12312^T^-inoculated kimchi, *Leuconostoc* (41.9%), *Bacillus* (10.1%), and *Lactobacillus* (8.1%) were the major genera on day 1. However, *Lactobacillus* became dominant from day 5 (36.6%) and remained so through day 20 (45.8%). In non-starter kimchi, *Bacillus* (28.6%), *Lactobacillus* (7.8%), *Vagococcus* (3.8%), *Leuconostoc* (3.1%), and *Weissella* (2.2%) were detected on day 1. By day 10, *Lactobacillus* became predominant (79.3%) and remained dominant on day 20 (73.8%) (Fig. 1B).

At the species level, in *Leu. lactis* DMLL10-inoculated kimchi, the Firmicutes population on day 1 mainly comprised *Leu. lactis* (54.1 %), *Bacillus subtilis* (6.7%), and *Latilactobacillus* (*Lb.*) *sakei* (4.1%). On day 5, *Leu. lactis* (63.1 %) and *Lb. sakei* (21.6%) were the most abundant, followed by *Leu. mesenteroides* (1.4 %). By day 20, *Leu. lactis* (60.5%), *Lb. sakei* (24.2%), and *Leu. gelidum* (7.5%) were identified, confirming that the inoculated *Leu. lactis* remained predominant throughout fermentation in the *Leu. lactis* DMLL10-inoculated kimchi. In contrast, in *Leu. mesenteroides* KACC 12312^T^-inoculated kimchi, *Leu. mesenteroides* (41.4%), *Lb. sakei* (7.8%), and *B. subtilis* (6.5%) were detected on day 1, but *Lb. sakei* became dominant from day 5 (36.6%) and maintained higher relative abundance (45.7%) than *Leu. mesenteroides* (37.4%) by day 20. Thus, although the inoculated *Leu. mesenteroides* strain remained prevalent during fermentation, *Lb. sakei* tended to become more dominant. In non-starter kimchi, *Lb. sakei* increased from 7.3% on day 1 to 73.7% on day 20, indicating that without a starter, *Lb. sakei* naturally becomes the predominant species, whereas starter inoculation effectively suppresses its relative abundance (Fig. 1B).

Unlike heat-treated fermented foods, kimchi is made from washed but raw vegetables, so fermentation is initiated by microorganisms originating from the raw materials. In this study, the microbial succession observed in non-starter kimchi reflected the natural microbiota derived from ingredients. In contrast, the inoculated strains (*Leu. lactis* DMLL10 and *Leu. mesenteroides* KACC 12312^T^) became dominant compared to the control (Fig. 1B). Notably, *Leu. lactis* DMLL10-inoculated kimchi showed a lower relative abundance of *Lb. sakei* than the control. Because *Lb. sakei* is a homo-fermentative LAB that produces two molecules of lactate from one glucose molecule—thereby increasing sourness—it is known as a late-stage fermenter that causes over-acidification of kimchi [11, 12]. Therefore, the lower relative abundance of *Lb. sakei* observed in the *Leu. lactis* DMLL10-inoculated kimchi suggests that this starter may have the potential to limit the relative expansion of late-stage LAB during fermentation. However, this interpretation should be considered with caution because the microbial community data in this study are based on relative abundance. Therefore, further studies using absolute quantification methods are needed to determine whether *Leu. lactis* DMLL10 directly suppresses the growth of *Lb. sakei* during kimchi fermentation.

It should also be noted that the predominance of *Lb. sakei* observed in non-starter kimchi may be due to methodological bias. As reported in our previous studies, 16S rRNA gene amplicon sequencing often indicates *Lb. sakei* dominance, whereas plate-culture analysis of the same samples reveals *Leu. mesenteroides* as the dominant species [3, 5, 26]. This discrepancy has been attributed to easier recovery and amplification of *Lb. sakei* DNA compared with *Leu. mesenteroides* DNA [4]. Thus, the dominant taxa and their proportions in kimchi microbiota may vary depending on the analytical method used. In this study, 16S rRNA gene amplicon sequencing was selected as a convenient approach to confirm starter dominance, and the results were sufficient to demonstrate that the inoculated starters were predominant. Nevertheless, if a reliable species- or strain-level tracking system for starters is established in the future, it could facilitate cost-effective industrial monitoring and maintenance of inoculated strains during kimchi fermentation.

### Effect of Organic Acid Production

Five organic acids—lactic acid, acetic acid, citric acid, propionic acid, and fumaric acid—were identified in kimchi samples from the three treatment groups (Fig. 2). Among them, lactic acid and acetic acid, which are the main fermentation products of kimchi, were not detected on day 1 but increased steadily as fermentation progressed (Fig. 2A).

In *Leu. lactis* DMLL10-inoculated kimchi, lactic acid increased more than threefold from 370.88 ± 2.78 mg/L on day 5 to 1326.61 ± 6.95 mg/L on day 20. Although the absolute values differed among treatments, a similar increasing pattern was observed in non-starter kimchi and *Leu. mesenteroides* KACC 12312^T^-inoculated kimchi. On day 20, lactic acid concentrations were 1543.39 ± 21.84 mg/L and 1291.79 ± 7.56 mg/L in the non-starter and *Leu. mesenteroides* KACC 12312^T^-inoculated kimchi, respectively, showing significant differences among all samples (Fig. 2A).

Acetic acid, known to be produced under aerobic conditions during hetero-lactic fermentation, also increased with fermentation time. In *Leu. lactis* DMLL10-inoculated kimchi, its concentration rose from 205.16 ± 4.36 mg/L on day 5 to 1035.30 ± 1.21 mg/L on day 20. The non-starter and *Leu. mesenteroides* KACC 12312^T^-inoculated kimchi showed similar trends, with respective day-20 concentrations of 829.27 ± 18.99 mg/L and 1045.83 ± 30.24 mg/L. No significant difference in acetic acid content was observed between the two starter-inoculated kimchi samples on day 20 (Fig. 2A).

Our previous study also demonstrated that lactate and acetate levels increase during kimchi fermentation, while citric acid decreases [27, 28]. In this study, citric acid likewise declined as fermentation progressed. In the non-starter kimchi, citric acid temporarily increased on day 5 before decreasing, consistent with earlier results. However, in both starter-inoculated kimchi samples, citric acid continued to decrease without an initial rise, showing a steeper decline in *Leu. lactis* DMLL10-inoculated kimchi and a more gradual decrease in *Leu. mesenteroides* KACC 12312^T^-inoculated kimchi (Fig. 2A).

Propionic acid is a minor organic acid in kimchi and is generally detected only during the later stages of fermentation [29]. Consistent with this, propionic acid was not detected during the early stages of fermentation but appeared in later stages in all three samples. In *Leu. lactis* DMLL10-inoculated kimchi, it was first detected on day 10 and increased significantly to 37.14 ± 5.91 mg/L by day 20. In contrast, propionic acid was detected only at day 20 in the non-starter and *Leu. mesenteroides* KACC 12312^T^-inoculated kimchi, at 30.41 ± 1.20 mg/L and 11.83 ± 1.51 mg/L, respectively, showing significant differences among the three groups (Fig. 2A).

Fumaric acid remained below 10 mg/L in all samples regardless of fermentation day, suggesting that it was not significantly affected by the starter. This low concentration was also consistent with previous kimchi studies [28]. Nonetheless, on day 1, *Leu. lactis* DMLL10-inoculated kimchi showed the highest value (8.06 ± 0.21 mg/L) (Fig. 2A).

A Pearson correlation heatmap was constructed to assess the relationship between fermentation days and organic acid production (Fig. 2B). Lactic acid and acetic acid showed negative correlations at day 1 in all samples but became positively correlated as fermentation progressed, with acetic acid showing a significant positive correlation in starter-inoculated kimchi. Citric acid displayed positive correlations immediately after fermentation began but turned negative during later stages, especially in *Leu. lactis* DMLL10-inoculated kimchi, where the negative correlation was significant. Propionic acid showed negative correlations until day 10 and positive correlations by day 20, with statistically significant positive relationships in both non-starter and *Leu. lactis*-inoculated kimchi. Fumaric acid generally showed negative correlations across all samples, although *Leu. lactis* DMLL10-inoculated kimchi showed a significant positive correlation on day 1 (Fig. 2B).

To investigate fermentation pattern based on organic acid production, PCA was performed using SPSS (Fig. 2C). PCA, an unsupervised multivariate statistical method, was applied to visualize the overall differences in organic acid profiles among kimchi samples according to starter culture and fermentation time. In the PCA score plot, each point represents an independent biological replicate, and the relative distance between points indicate the degree of similarity or dissimilarity in organic acid composition among the samples. In the PCA loading plot, lactic acid, acetic acid, and propionic acid were positioned on the positive side of PC1, while citric acid and fumaric acid were located on the negative side (Fig. S1A). On day 1, *Leu. lactis* DMLL10-inoculated kimchi was positioned in the second quadrant, moved to the third quadrant on day 5, and then shifted toward the first quadrant as fermentation proceeded. *Leu. mesenteroides* KACC 12312^T^-inoculated kimchi began in the third quadrant but followed a similar trajectory, eventually clustering in the first quadrant. The non-starter kimchi showed a comparable trend, shifting from the third to the second quadrant on day 5 and then to the first quadrant by day 20 (Fig. 2C). The positioning of *Leu. lactis* DMLL10-inoculated kimchi on day 1 and non-starter kimchi on day 5 in the second quadrant was likely due to relatively higher fumaric acid levels; however, since fumaric acid did not differ significantly among samples, this variation may reflect differences in sample extraction rather than fermentation pattern.

To further assess the separation observed in the PCA, OPLS-DA analysis was performed using SIMCA. As a supervised multivariate analysis, OPLS-DA was applied to evaluate whether the organic acid profiles could discriminate kimchi samples according to starter culture and fermentation time. Although the loading orientations (vertical/horizontal orientation) differed from those of the PCA model, the overall clustering pattern was highly consistent between the two analyses (Fig. 2C and Fig. S1A).

Taken together, all kimchi samples exhibited a similar fermentation pattern characterized by increasing levels of lactic, acetic, and propionic acids and decreasing citric acid over time. However, the degree of change varied depending on the inoculated strain, suggesting that different starters may influence kimchi quality and sensory characteristics through distinct organic acid production profiles.

### Effect of Amino Acid Production

Amino acids are key contributors to the flavor characteristics of kimchi [30, 31]. Therefore, amino acid composition was analyzed to evaluate the impact of starter inoculation on the taste-related properties of fermented kimchi (Table 1). The changes in amino acid content during fermentation differed among the three sample groups.

In *Leu. lactis* DMLL10-inoculated kimchi, levels of alanine, γ-aminobutyric acid (GABA), glutamic acid, glycine, isoleucine, leucine, lysine, methionine, phenylalanine, threonine, tryptophan, tyrosine, and valine increased as fermentation progressed, while aspartic acid and taurine decreased. The decrease in aspartic acid was consistent with our previous results [27], confirming its typical decline during kimchi fermentation. Other amino acids showed no clear, consistent trends over time. A similar pattern of amino acid variation was observed in *Leu. mesenteroides* KACC 12312^T^-inoculated kimchi, although glutamine showed a distinct increase. Overall, *Leu. lactis* DMLL10-inoculated kimchi tended to show higher levels of several amino acids during fermentation, particularly at the later fermentation stage. However, the day 1 samples should be considered an early fermentation point rather than true pre-fermentation baseline samples, and therefore early-stage differences should be interpreted with caution (Table 1). In addition, because the first sampling point was day 1, the initial equivalence of the mixed raw-material matrix among the treatment groups could not be directly assessed, which represents a limitation of this study. Furthermore, because the starter cultures were inoculated as culture broth rather than as washed cell suspensions, the possible contribution of inoculum-derived metabolites or medium components to the day 1 amino acid profiles cannot be excluded. Therefore, the amino acid results should be interpreted as observed amino acid profile differences during fermentation, rather than as definitive evidence of starter-specific amino acid production within the kimchi matrix.

To examine correlations between fermentation time and amino acid production, Pearson correlation coefficients were visualized in a heatmap (Fig. 3A). In *Leu. lactis* DMLL10-inoculated kimchi, correlations shifted from negative to positive as fermentation progressed, with 15 of the 20 detected amino acids showing strong, statistically significant positive correlations by day 20. In contrast, *Leu. mesenteroides* KACC 12312^T^-inoculated kimchi showed significant negative correlations for 14 amino acids on day 1, which gradually shifted to positive values during fermentation. However, only three amino acids—proline, asparagine, and arginine—showed significant positive correlations by day 20. The non-starter kimchi exhibited a similar trend to *Leu. mesenteroides* KACC 12312^T^-inoculated kimchi, but on day 20, four amino acids (leucine, methionine, phenylalanine, and lysine) showed significant positive correlations. Interestingly, at day 10, four amino acids—asparagine, aspartic acid, glutamic acid, and arginine—displayed significant negative correlations (Fig. 3A). These findings indicate that the tested starter strains were associated with different temporal patterns of amino acid production during kimchi fermentation. In particular, glutamic acid—well known for imparting umami taste [32] —was produced at the highest concentration on day 20 in *Leu. lactis* DMLL10-inoculated kimchi. The higher glutamic acid level in *Leu. lactis* DMLL10-inoculated kimchi may contribute to umami-related taste. Nevertheless, because sensory evaluation was beyond the scope of this study, this conclusion should be considered a metabolite-based interpretation rather than direct evidence of enhanced sensory quality. Future studies integrating metabolomic and sensory analyses will be necessary to determine whether these changes in glutamic acid and other flavor-related metabolites are reflected in the perceived sensory quality of kimchi.

To further explore fermentation patterns based on amino acid production, PCA analysis was performed (Fig. 3B). Each point in the score plot represents an independent replicate, and the distance among points reflects differences in amino acid composition. In the PCA loading plot, aspartic acid and taurine were positioned on the negative side of PC1, while all other amino acids were on the positive side (Fig. S1B). On day 1, *Leu. lactis* DMLL10-inoculated kimchi was positioned on the positive side of PC1, distinct from the other samples. It shifted to the fourth quadrant on day 5 and then moved toward the first quadrant as fermentation advanced. *Leu. mesenteroides* KACC 12312^T^-inoculated kimchi followed a similar pattern but started on the negative side of PC1 on day 1 before shifting from the fourth to the first quadrant as fermentation progressed. The non-starter kimchi was located in the negative region of both PC1 and PC2 on day 1 and moved to the fourth quadrant by day 20 (Fig. 3B).

To validate the PCA results, OPLS-DA analysis was conducted, showing that although the loading axes were inverted vertically and horizontally, the overall fermentation trends were consistent with the PCA data (Fig. 3B and Fig. S1B). These results indicate that amino acid profiles changed progressively during fermentation and that the temporal amino acid patterns differed among the kimchi samples.

In summary, amino acid production patterns varied depending on the starter culture. *Leu. lactis* DMLL10-inoculated kimchi contained relatively high amino acid levels from the early fermentation stage and showed greater amino acid accumulation than the non-starter kimchi as fermentation proceeded. These results suggest that different starters influence the generation of taste-related amino acids in distinct ways, thereby contributing differently to the sensory characteristics and flavor complexity of fermented kimchi.

### Effect of Volatile Compound Production

Volatile compounds shape the aroma of foods and enhance flavor complexity through diverse chemical constituents such as acids, aldehydes, alcohols, esters, and sulfur compounds. Consequently, they represent one of the most critical factors influencing consumer acceptability [30, 33]. To assess how starter inoculation influences the formation of volatile aroma compounds during kimchi fermentation, headspace SPME–GC–MS analyses were performed by fermentation day (Fig. 4 and Table S2). In kimchi inoculated with *Leu. lactis* DMLL10, seven volatile compounds were detected immediately after mixing (day 1), and additional acids, alcohols and aldehydes appeared by day 10. A similar increase in acids and alcohols was observed on day 10 in *Leu. mesenteroides* KACC 12312^T^-inoculated kimchi. In the non-starter kimchi, acids were first detected on day 20, and the number of alcohols detected was lower than in starter-inoculated kimchi. These results indicate that *Leu. lactis* DMLL10 promotes a richer volatile profile (Fig. 4A).

Beyond the number of volatiles, their total abundance also changed: in *Leu. lactis* DMLL10–inoculated kimchi, total volatile content peaked on day 10. The non-starter kimchi showed the same pattern, whereas *Leu. mesenteroides* KACC 12312^T^-inoculated kimchi displayed little increase relative to day 1 (Fig. 4A). Consistent with earlier reports, overall volatile abundance tended to be higher around day 10 than day 1 and then gradually declined. [7] Notably, aldehydes in *Leu. lactis* DMLL10-inoculated kimchi increased in both number and amount on day 10 and then decreased thereafter (Fig. 4A).

We next examined individual volatiles and analyzed correlations with fermentation time using Pearson correlation coefficients (Fig. 5 and Table S2). In *Leu. lactis* DMLL10-inoculated kimchi, the acids detected were acetic acid and octanoic acid; neither was present on day 1, but both appeared on days 10 and 20. Octanoic acid was detected only in the *Leu. lactis* DMLL10-inoculated kimchi, showing a positive correlation with fermentation time, and was absent from non-starter and *Leu. mesenteroides* KACC 12312^T^-inoculated kimchi—suggesting a specific contribution of strain DMLL10 to octanoic acid formation. In *Leu. mesenteroides* KACC 12312^T^-inoculated kimchi, acetic acid was detected from day 10 and was significantly higher than in the other samples by day 20, exhibiting a positive correlation (Fig. 5 and Table S2).

Among alcohols, most compounds—except 3,7-dimethylocta-1,6-dien-3-ol and (2E)-3,7-dimethylocta-2,6-dien-1-ol—first appeared on day 10 and reached their highest levels in *Leu. lactis* DMLL10-inoculated kimchi on that day. Seven alcohols were detected as fermentation progressed: (2Z)-pent-2-en-1-ol, hexan-1-ol, (E)-hex-2-en-1-ol, 6-methylhept-5-en-2-ol, 4-(1-methylethyl)-1-cyclohexen-1-ol, (E)-oct-2-en-1-ol, and nonan-1-ol (Table S2). Of note, (2Z)-pent-2-en-1-ol and (E)-oct-2-en-1-ol showed positive correlations only in *Leu. lactis* DMLL10-inoculated kimchi, whereas 3,7-dimethylocta-1,6-dien-3-ol and 4-(1-methylethyl)-1-cyclohexen-1-ol showed positive correlations only in *Leu. mesenteroides* KACC 12312^T^-inoculated kimchi, indicating strain-specific contributions to these alcohols (Fig. 5 and Table S2).

Five aldehydes—(2E)-hept-2-enal, (2E,4E)-hepta-2,4-dienal, (2E,6Z)-nona-2,6-dienal, (2Z)-3,7-dimethylocta-2,6-dienal, and (E)-3,7-dimethylocta-2,6-dienal—were identified. Among these, (2E)-hept-2-enal, (2E,4E)-hepta-2,4-dienal, and (2E,6Z)-nona-2,6-dienal were absent on day 1 but appeared from day 10, showing significantly higher levels and positive correlations in *Leu. lactis* DMLL10-inoculated kimchi. Particularly, (2E,6Z)-nona-2,6-dienal were detected only in *Leu. lactis* DMLL10-inoculated kimchi, implying that this strain likely participates in their formation (Fig. 5 and Table S2).

Four isothiocyanates were detected. Interestingly, *Leu. lactis* DMLL10-inoculated kimchi showed no correlation or negative correlations with fermentation time, whereas *Leu. mesenteroides* KACC 12312^T^-inoculated kimchi displayed generally positive correlations up to day 10, which then disappeared (Fig. 5 and Table S2). Isothiocyanates—responsible for pungent notes—are typically abundant early in fermentation and decline thereafter [34], a trend also observed here.

Ketones were notable in that two compounds—6-methylhept-5-en-2-one and undecan-2-one—were present at significant levels only in non-starter kimchi; no meaningful correlations were observed in starter-inoculated samples (Fig. 5 and Table S2).

Nitriles showed positive correlations on day 1 that disappeared thereafter. As an exception, pent-4-enenitrile exhibited a positive correlation at day 10 in the starter-inoculated groups but decreased thereafter. Overall, nitriles did not differ significantly among treatments and appear to be common constituents of kimchi regardless of the starter used (Fig. 5 and Table S2).

Sulfur compounds constituted the largest class of volatiles detected. These compounds—derived from key ingredients such as *baechu*, chili, garlic, green onion, and ginger—are well documented in kimchi [35]. In total, 23 sulfur compounds were identified; 12 of them were found across all samples irrespective of starter inoculation or fermentation day. However, ethene sulfide, methyl thiocyanate, hexahydropyrrolizine-3-thione, and dipropyl trisulfide varied significantly with fermentation day: ethene sulfide and hexahydropyrrolizine-3-thione disappeared as fermentation progressed, whereas methyl thiocyanate and dipropyl trisulfide first appeared from days 5 and 10, respectively (Table S2). Pearson correlation analysis showed broadly positive correlations at day 10 in *Leu. lactis* DMLL10-inoculated kimchi and non-starter kimchi, whereas *Leu. mesenteroides* KACC 12312^T^-inoculated kimchi generally had lower levels and showed no significant correlations (Fig. 5). Although sulfur compounds are often attributed to seasoning ingredients, the reduction observed with certain starters suggests a substantial microbial influence, warranting further investigation.

In the terpene class, four volatiles did not show clear patterns by fermentation day or starter treatment. However, (6Z,10Z)-3,7,11-trimethyldodeca-1,6,10-triene was detected only on days 5 and 10 in non-starter kimchi, showing positive correlations, and (1S,5S)-2-methyl-5-((R)-6-methylhept-5-en-2-yl)bicyclo[3.1.0]hex-2-ene showed a positive correlation only on day 10 in non-starter kimchi (Fig. 5 and Table S2). Although terpenes are reported to be abundant early in fermentation and to decline after ~30 days [36], our 20-day fermentation window likely limited major differences. Moreover, sesquiterpenes have relatively high sensory thresholds and may contribute less to kimchi flavor than sulfur compounds [36].

To visualize starter-dependent differences in volatile classes and amounts, PCA was performed (Fig. 4B). Each point in the score plot represents an independent replicate, and the distance among points reflects differences in volatile compound profiles. Among the eight categories, sulfur compounds were broadly distributed but mainly located on the positive side of PC1 (Fig. S1C). On day 1, volatile profiles from all kimchi samples clustered in the third quadrant. On day 10, both *Leu. lactis* DMLL10-inoculated kimchi and non-starter kimchi shifted to the fourth quadrant, driven by increased sulfur compounds, whereas *Leu. mesenteroides* KACC 12312^T^-inoculated kimchi moved to the first or second quadrant. By day 20, all samples shifted to the first or second quadrant (positive PC2), reflecting increased acids and alcohols, with stronger positive associations in the starter-inoculated kimchi (Fig. 4B). These results indicate that volatile compound profiles changed during fermentation and that the pattern of change differed among the kimchi samples.

OPLS-DA was used to validate the PCA. Compared with PCA, compound positions and score plots were mirrored left–right, but overall trends were consistent (Fig. 4B and Fig. S1C). OPLS-DA further clarified differences on day 20 associated with increased acids and alcohols.

Overall, the effect of starter inoculation on volatile compound profiles was minimal during the stage of fermentation but became increasingly evident from day 10 onward, with distinct differences in both compound composition and relative abundance. Given the important role of volatile compounds in determining kimchi aroma, these findings indicate that development of volatile characteristics is influenced by the starter culture used. However, volatile compound abundance alone does not necessarily predict sensory quality because odor-active compounds differ substantially in their odor thresholds, odor descriptors, and contributions to the overall aroma. Accordingly, the observed differences should be regarded as metabolomic evidence of starter-dependent variation in aroma-related compounds rather than direct evidence of enhanced sensory properties. Further studies combining odor activity value analysis with sensory evaluation are required to clarify whether these metabolomic differences translate into perceptible changes in aroma and overall sensory quality.

### Network-Based Comparison of Metabolite Dynamics Between Starter Culture Type

Based on the above results, a comprehensive analysis of metabolic differences according to starter inoculation was conducted using Pearson correlation coefficients for organic acids, amino acids, and volatile compounds, visualized through Cytoscape (Fig. 6). In the network plots, fermentation days and metabolites are shown as nodes, and the connecting lines indicate statistically significant Pearson correlations. For volatile compounds, only acids, alcohols, and aldehydes—those most likely influenced by starter inoculation—were analyzed. When comparing metabolite production between the two strains, kimchi inoculated with *Leu. lactis* DMLL10 showed positive correlations between fumaric acid and several amino acids on day 1, but predominantly negative correlations on day 5. By day 10, positive correlations appeared between amino acids and various volatile compounds (excluding organic acids), and by day 20, the number of positively correlated organic acids, volatiles, and amino acids further increased. In contrast, *Leu. mesenteroides* KACC 12312^T^-inoculated kimchi exhibited fewer positively correlated metabolites across days 1, 5, and 10, with the number increasing by day 20—though still fewer than those observed in the *Leu. lactis* DMLL10-inoculated kimchi (Fig. 6A).

When visualized by sample groups (Fig. 6B), the correlation networks among metabolites clearly differed among non-starter kimchi, *Leu. lactis* DMLL10-inoculated kimchi, and *Leu. mesenteroides* KACC 12312^T^-inoculated kimchi. These findings provide scientific evidence that the inoculated starter type alters the metabolite composition during kimchi fermentation. Although both strains belong to the *Leuconostoc* genus, their distinct metabolic outputs indicate that the type of starter strain can influence the sensory profile and potentially consumer preference. However, it remains to be determined whether these differences arise at the species or strain level. Further studies involving multiple strains and sensory evaluations of the final fermented products are therefore recommended.

The choice of food products has evolved in parallel with societal and economic shifts. Before the 1990s, food production primarily aimed to alleviate hunger through increased yields. In the 1990s, consumer interest shifted toward functional foods, and by the 2000s, the diversification of consumer demands led to the era of personalized nutrition. Similarly, the fermented food industry now faces the need to produce customized fermented products. This requires a more precise understanding of the microorganisms involved in fermentation and their specific contributions to metabolite formation. Kimchi is one of the most widely consumed fermented foods in Korea, and *Leu. mesenteroides* has traditionally been used as the principal starter culture. However, reliance on a single starter strain has contributed to the uniformity and simplification of commercial kimchi. To overcome this limitation, the application of diverse starter strains is needed to promote product diversification and premiumization. In this context, the present study demonstrates that *Leu. lactis* produces a metabolite profile distinct from *Leu. mesenteroides*, suggesting that the use of *Leu. lactis* as a starter may serve as a valuable foundation for developing more diverse and differentiated kimchi products in the future.

## Supplemental Materials

Supplementary data for this paper are available on-line only at http://jmb.or.kr.



## Figures and Tables

**Fig. 1:**
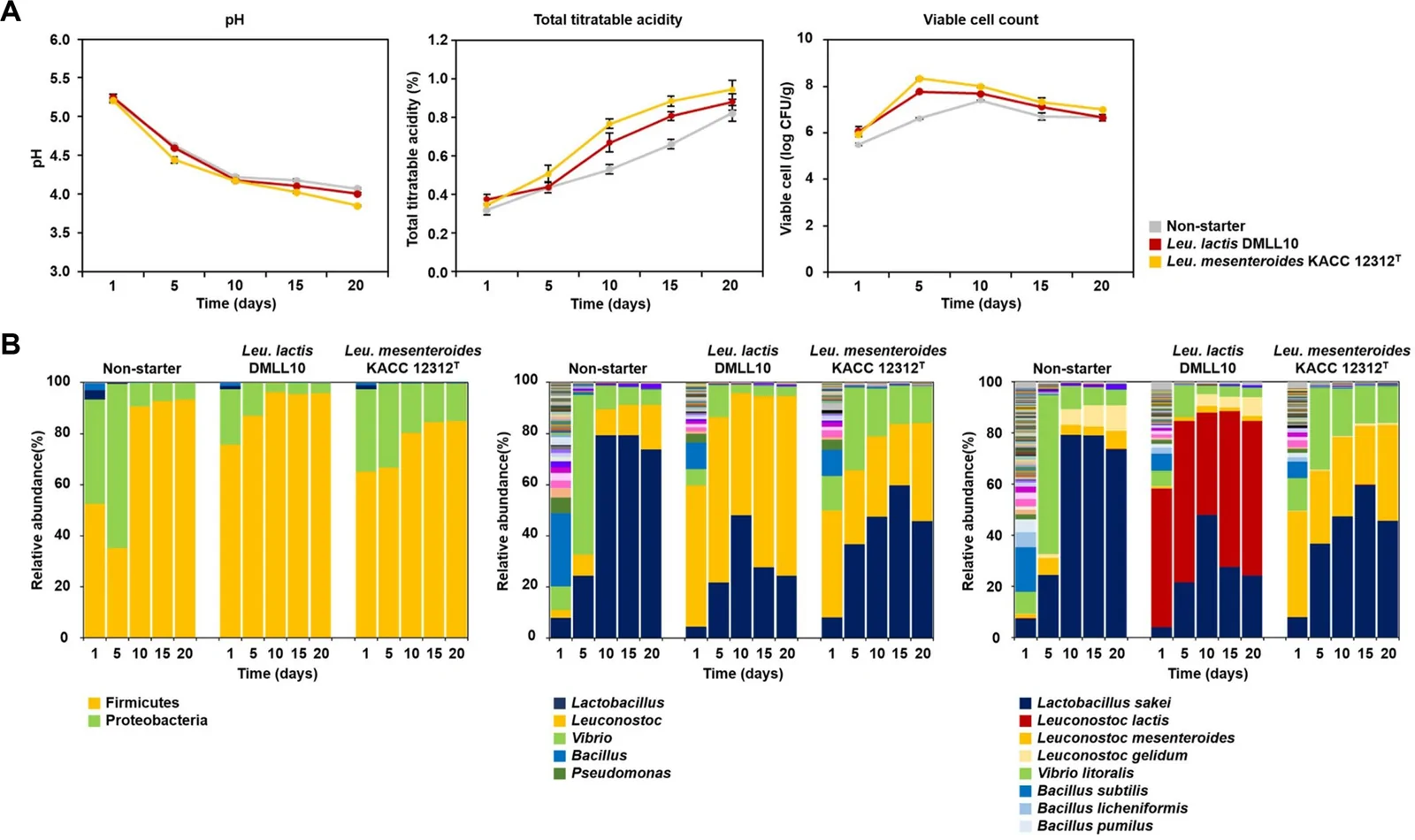
Changes in physicochemical properties (A) and bacterial community dynamics (B) during kimchi fermentation at 4°C. (**A**) pH, total titratable acidity, and viable cell counts were determined independently in triplicate, and the mean values of the replicates are presented. (**B**) Bacterial community dynamics based on culture-independent analysis at the phylum, genus, and species levels. Sequences longer than 200 bp were clustered using CD-HIT-OUT with a 97% similarity threshold, and only categories with a relative abundance greater than 5% are shown.

**Fig. 2:**
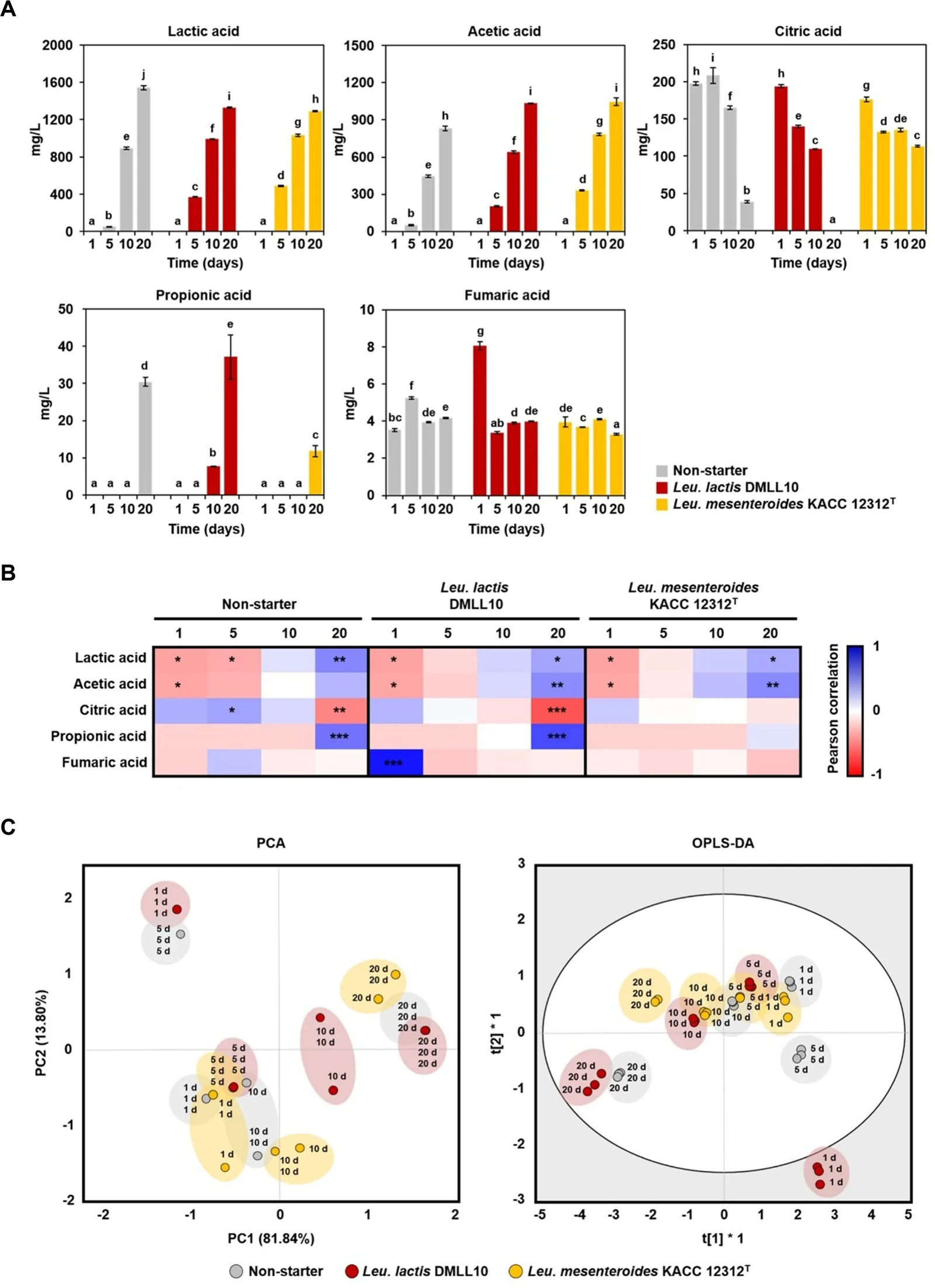
Changes in organic acids according to starter culture addition. (**A**) Changes in organic acid content during fermentation. Organic acid concentrations were expressed as mg/L of the aqueous extract. All values were determined independently in triplicate, and the mean values of the replicates are presented. Different letters indicate significant differences at *p* < 0.05 according to Duncan’s multiple range test. (**B**) Pearson correlation coefficient heatmap between starter culture addition and organic acid production. Red indicates negative correlations, while blue indicates positive correlations. Asterisks denote statistical significance at *P* < 0.05, *P* < 0.01, and *P* < 0.001 (*, **, ***). (**C**) PCA and OPLS-DA score plots based on starter culture addition and organic acid production. Each point represents an independent replicate for each fermentation day and treatment group, and the relative distance among points indicates differences in organic acid profiles.

**Fig. 3:**
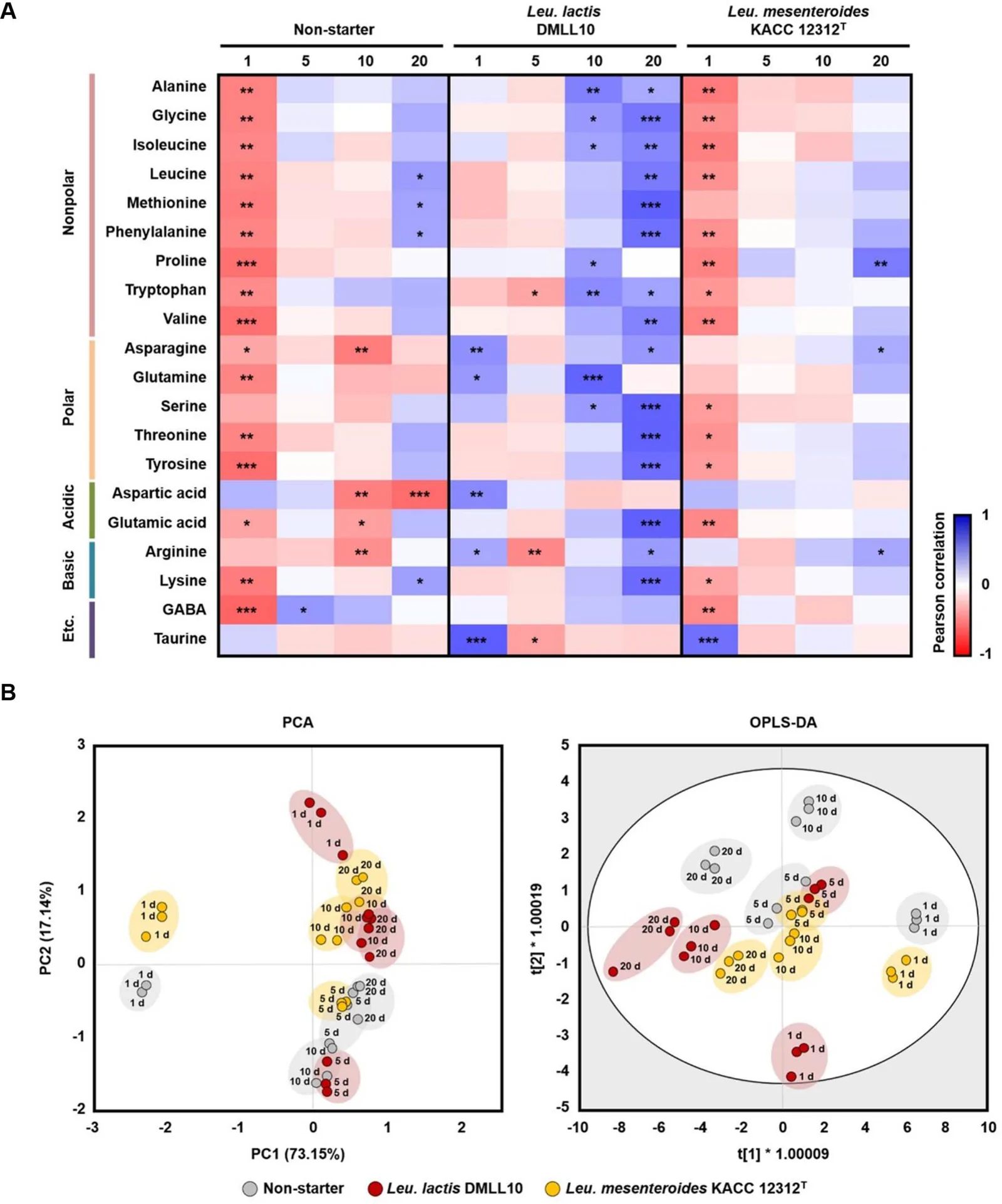
Changes in amino acids according to starter culture addition. (**A**) Pearson correlation coefficient heatmap between starter culture addition and amino acid production. Red indicates negative correlations, while blue indicates positive correlations. Asterisks denote statistical significance at *P* < 0.05, *P* < 0.01, and *P* < 0.001 (*, **, ***). (**B**) PCA and OPLS-DA score plots based on starter culture addition and amino acid production. Each point represents an independent replicate for each fermentation day and treatment group, and the relative distance among points indicates differences in amino acid profiles.

**Fig. 4:**
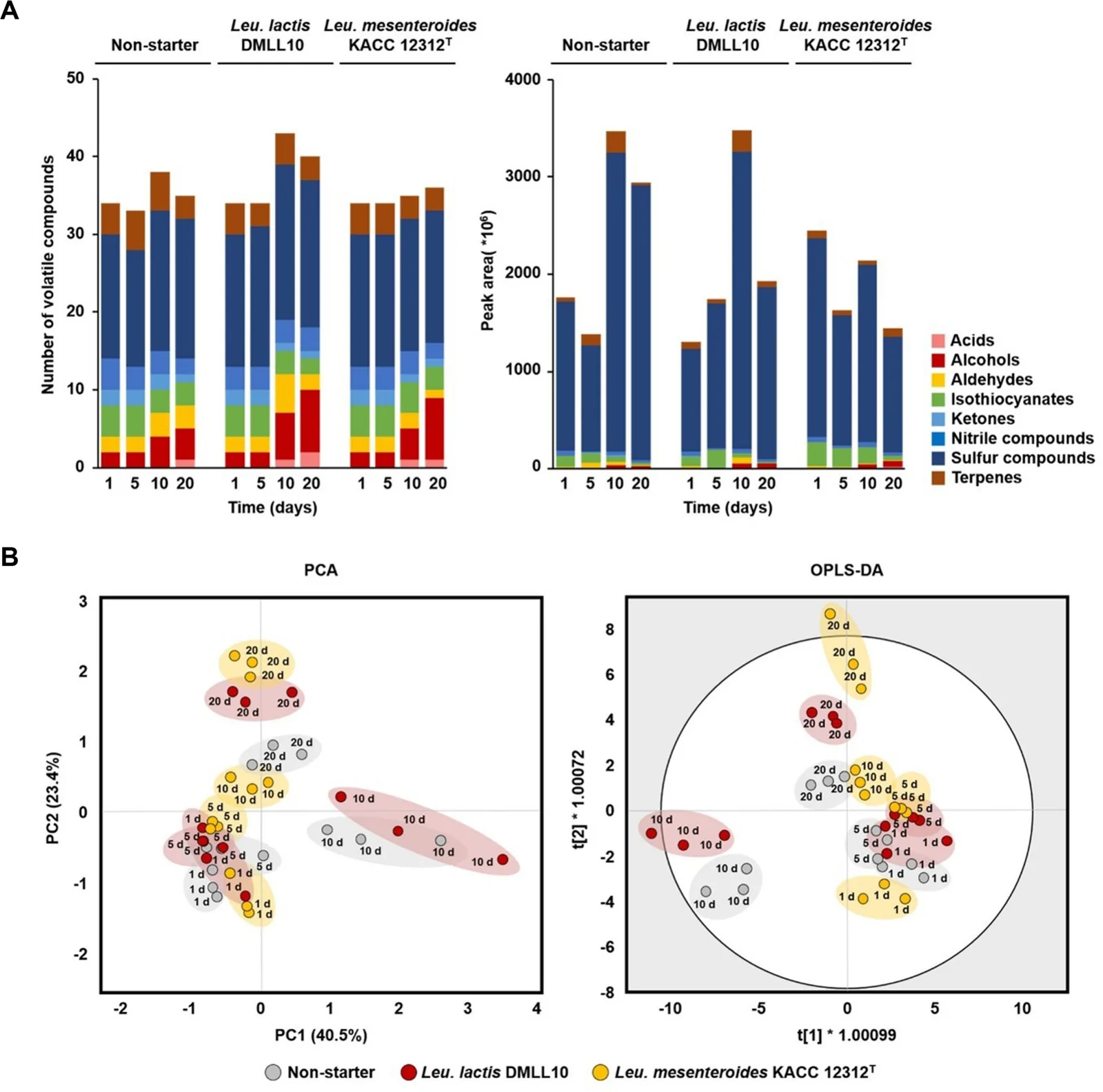
Changes in the production of volatile compounds according to starter culture addition. (**A**) The number of volatile compounds and the changes in their contents were determined independently in triplicate, and the mean values of the replicates are presented. (**B**) PCA and OPLS-DA score plots based on starter culture addition and volatile compound production. Each point represents an independent replicate for each fermentation day and treatment group, and the relative distance among points indicates differences in volatile compounds profiles.

**Fig. 5:**
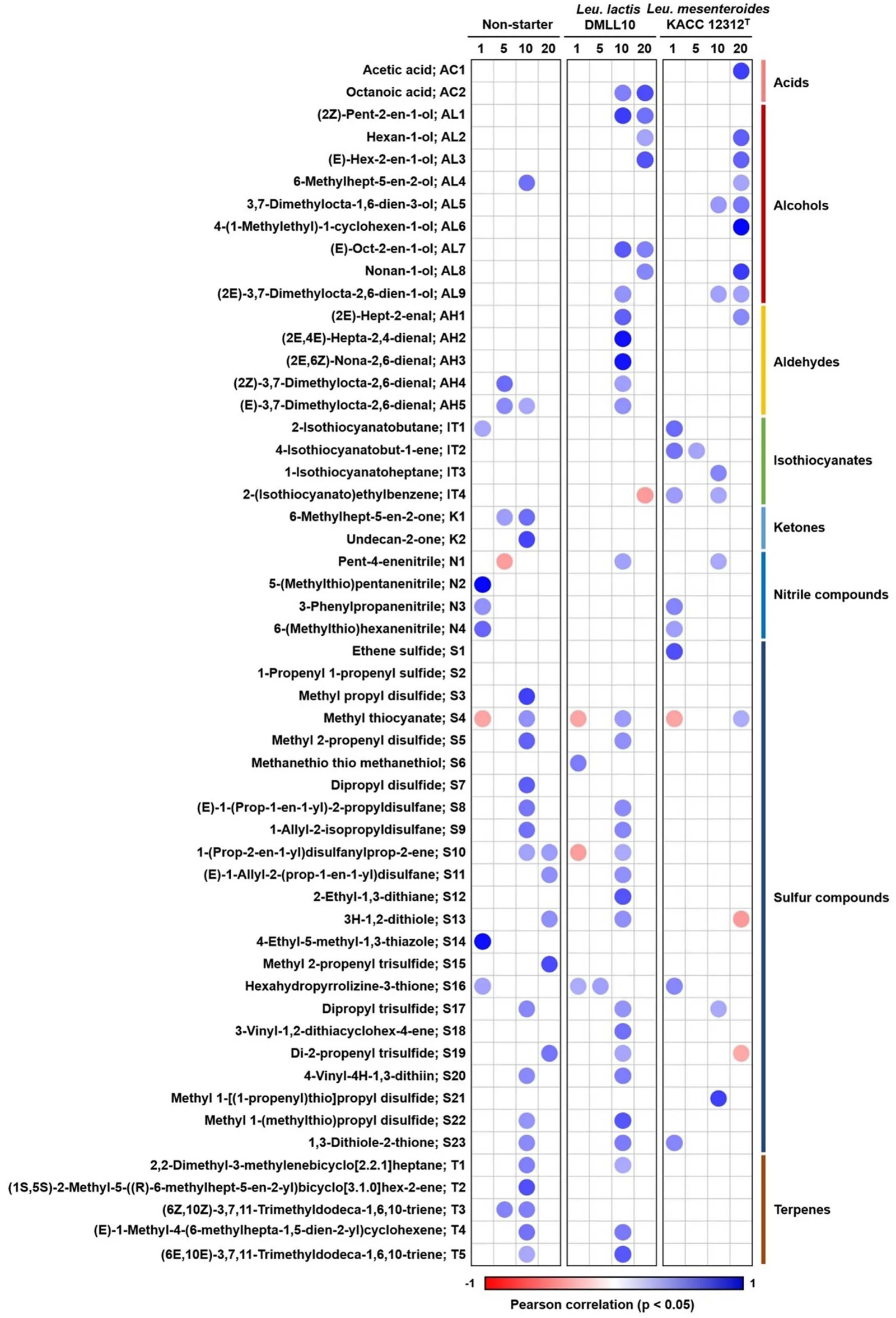
Pearson correlation coefficients between starter cultures and volatile compound production. Red indicates negative correlations, and blue indicates positive correlations. All values exhibit statistical significance at *P* < 0.05.

**Fig. 6:**
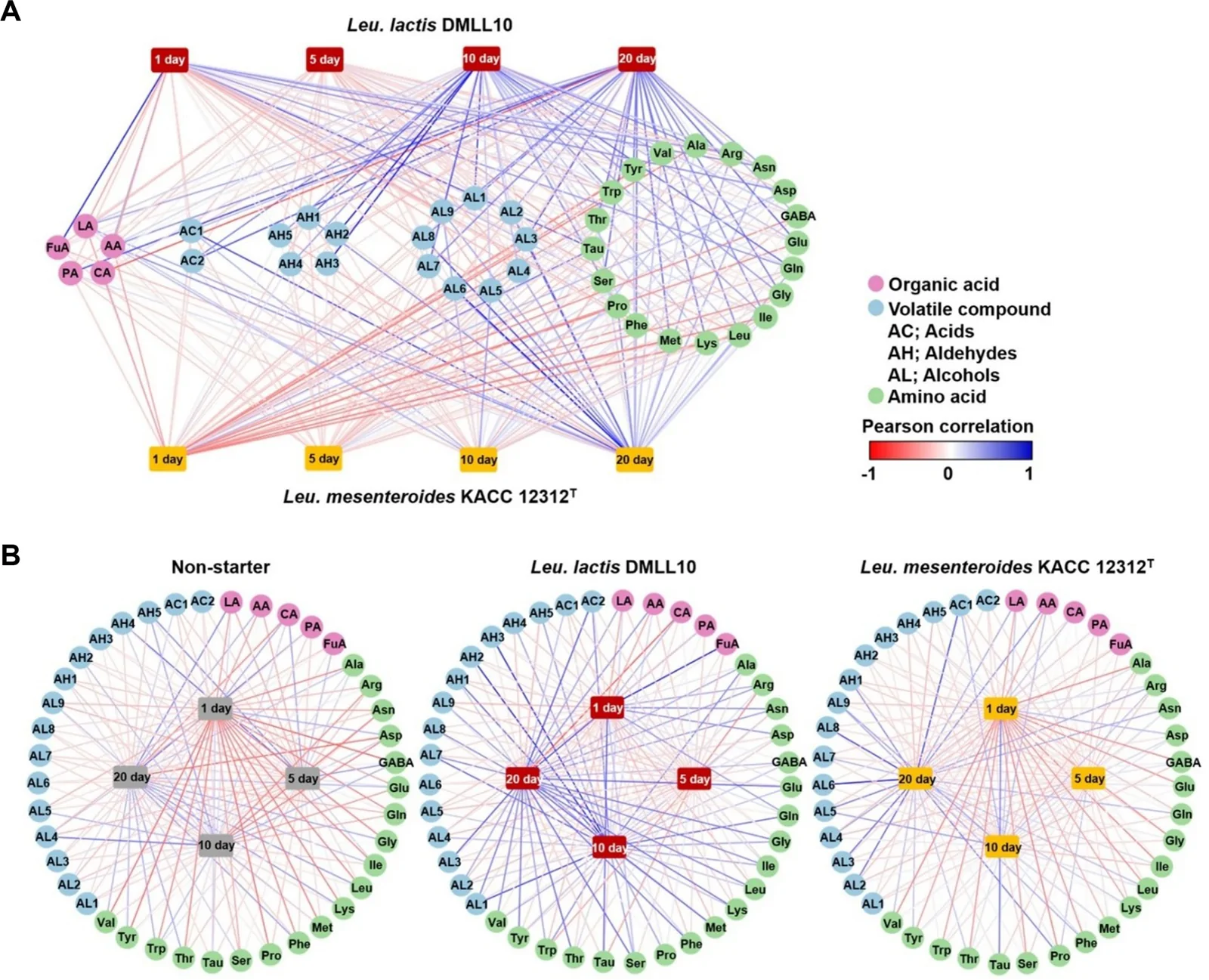
Cytoscape network analysis of fermentation metabolites based on starter culture addition (A) and kimchi sample (B). Squares represent fermentation days, and circles indicate fermentation metabolites. Each line connecting two nodes represents a Pearson correlation coefficient. Red indicates negative correlations, while blue indicates positive correlations.

**Table 1:** Changes of amino acid content during kimchi fermentation.