# Supplementary material for: Comparative Effects of Leuconostoc lactis and Leuconostoc mesenteroides Starters on Kimchi Fermentation Dynamics and Metabolite Profiles
Source: J Microbiol Biotechnol. 2026 Sep 23;36:e2609002. doi: 10.4014/jmb.2609.09002 (PMC13655590; doi:10.4014/jmb.2609.09002)
Supplement: Supplementary file 1 [file jmb-36-e2609002-supple.pdf]

J. Microbiol. Biotechnol. 2026. 36: e2609002
https://doi.org/10.4014/jmb.2609.09002

Supplementary Tables and Figure

Comparative Effects of *Leuconostoc lactis* and *Leuconostoc mesenteroides* Starters on Kimchi Fermentation Dynamics and Metabolite Profiles

Yura Moon[1], Sojeong Heo[1], Gawon Lee[1], Junghyun Park[1], Seung-Eun Oh[1,2], Sumin Lee[1], Minkyeong Kim[1], and Do-Won Jeong[1*]

[1]*Department of Food and Nutrition, Dongduk Women's University, Seoul 02748, Republic of Korea*

[2]*Dairy Products Team, Product Development Center, R&BD Division, hy Co., Ltd., Yongin-si, Gyeonggi-do, 17086, Republic of Korea*

Table S1. Changes of pH, total titratable acidity, and viable cell counts during kimchi fermentation.

	Starter	1 day	5 day	10 day	15 day	20 day
Changes of pH	Non-starter	5.22 ± 0.02^{i}	4.63 ± 0.02^{h}	4.23 ± 0.02^{f}	4.18 ± 0.02^{e}	4.07 ± 0.01^{c}
	Leu. lactis DMLL10	5.25 ± 0.04^{j}	4.60 ± 0.01^{h}	4.18 ± 0.01^{e}	4.11 ± 0.01^{d}	4.01 ± 0.01^{b}
	Leu. mesenteroides KACC 12312^{T}	5.21 ± 0.03^{i}	4.44 ± 0.04^{g}	4.17 ± 0.01^{e}	4.03 ± 0.02^{b}	3.85 ± 0.01^{a}
Changes of total titratable acidity (%)	Non-starter	0.32 ± 0.03^{a}	0.43 ± 0.03^{b}	0.53 ± 0.02^{c}	0.66 ± 0.02^{d}	0.82 ± 0.04^{e}
	Leu. lactis DMLL10	0.37 ± 0.02^{a}	0.44 ± 0.00^{b}	0.67 ± 0.05^{d}	0.81 ± 0.02^{e}	0.88 ± 0.04^{f}
	Leu. mesenteroides KACC 12312^{T}	0.35 ± 0.00^{a}	0.51 ± 0.04^{c}	0.77 ± 0.02^{e}	0.88 ± 0.03^{f}	0.94 ± 0.05^{g}
Changes of viable cell counts (Log CFU/g)	Non-starter	3.28 ± 0.07^{a}	6.48 ± 0.05^{d}	7.33 ± 0.11^{f}	6.41 ± 0.04^{d}	6.15 ± 0.05^{c}
	Leu. lactis DMLL10	$6.01\pm0.05^{b,c}$	7.62 ± 0.01^{g}	$7.46\pm0.05^{f,g}$	6.91 ± 0.01^{e}	6.46 ± 0.07^{d}
	Leu. mesenteroides KACC 12312^{T}	5.95 ± 0.01^{b}	8.28 ± 0.04^{h}	8.13 ± 0.09^{h}	7.31 ± 0.02^{f}	6.88 ± 0.26^{e}

All values were determined three times independently and the mean values of the replicates are presented. Different letter in each column indicates significant difference at $p < 0.05$ using Duncan's multiple range test.

Table S2. Semi-quantitative changes in volatile compounds during kimchi fermentation.

Volatile compounds (ppm Methyl cinnamate equivalents)	Abbreviation	RT	RI	Non-starter				*Leuconostoc lactis* DMLL10				*Leuconostoc mesenteroides* KACC 12312^{T}			
				1 day	5 day	10 day	20 day	1 day	5 day	10 day	20 day	1 day	5 day	10 day	20 day
Acids															
Acetic acid	AC1	25.97	1449	0.00±0.00^{a}	0.00±0.00^{a}	0.00±0.00^{a}	6.76±3.29^{b}	0.00±0.00^{a}	0.00±0.00^{a}	0.00±0.00^{a}	8.44±4.72^{b}	0.00±0.00^{a}	0.00±0.00^{a}	9.09±3.20^{b}	18.56±3.75^{c}
Octanoic acid	AC2	58.54	2060	0.00±0.00^{a}	0.00±0.00^{a}	0.00±0.00^{a}	0.00±0.00^{a}	0.00±0.00^{a}	0.00±0.00^{a}	1.08±0.36^{b}	1.42±0.89^{b}	0.00±0.00^{a}	0.00±0.00^{a}	0.00±0.00^{a}	0.00±0.00^{a}
Alcohols															
(2Z)-Pent-2-en-1-ol	AL1	18.43	1318	0.00±0.00^{a}	0.00±0.00^{a}	0.00±0.00^{a}	0.00±0.00^{a}	0.00±0.00^{a}	0.00±0.00^{a}	5.85±0.89^{c}	4.51±0.50^{b}	0.00±0.00^{a}	0.00±0.00^{a}	0.00±0.00^{a}	0.00±0.00^{a}
Hexan-1-ol	AL2	20.26	1355	0.00±0.00^{a}	0.00±0.00^{a}	4.81±0.76^{b,c}	4.65±0.91^{b,c}	0.00±0.00^{a}	0.00±0.00^{a}	5.67±0.73^{b,c}	8.27±0.70^{c}	0.00±0.00^{a}	0.00±0.00^{a}	3.70±2.12^{a,b}	11.99±7.00^{d}
(E)-Hex-2-en-1-ol	AL3	23.39	1405	0.00±0.00^{a}	0.00±0.00^{a}	0.00±0.00^{a}	0.00±0.00^{a}	0.00±0.00^{a}	0.00±0.00^{a}	0.00±0.00^{a}	1.81±0.17^{b}	0.00±0.00^{a}	0.00±0.00^{a}	0.00±0.00^{a}	1.67±0.75^{b}
6-Methylhept-5-en-2-ol	AL4	26.91	1465	0.00±0.00^{a}	0.00±0.00^{a}	3.29±1.57^{d}	1.80±0.30^{b,c}	0.00±0.00^{a}	0.00±0.00^{a}	2.04±0.61^{b,c}	1.26±0.19^{b}	0.00±0.00^{a}	0.00±0.00^{a}	1.26±0.55^{b}	2.46±0.97^{c,d}
3,7-Dimethylocta-1,6-dien-3-ol	AL5	31.82	1547	2.50±0.10^{a}	5.56±2.90^{a}	12.83±4.45^{b,c}	5.91±2.94^{a}	3.42±1.32^{a}	2.65±0.50^{a}	11.30±5.78^{b}	4.83±1.42^{a}	4.03±0.89^{a}	5.23±0.01^{a}	14.91±5.04^{b,c}	17.32±4.98^{c}
4-(1-Methylethyl)-1-cyclohexen-1-ol	AL6	34.17	1602	0.00±0.00^{a}	0.00±0.00^{a}	0.00±0.00^{a}	0.00±0.00^{a}	0.00±0.00^{a}	0.00±0.00^{a}	0.00±0.00^{a}	0.00±0.00^{a}	0.00±0.00^{a}	0.00±0.00^{a}	0.00±0.00^{a}	1.66±0.34^{b}
(E)-Oct-2-en-1-ol	AL7	35.36	1614	0.00±0.00^{a}	0.00±0.00^{a}	0.00±0.00^{a}	0.00±0.00^{a}	0.00±0.00^{a}	0.00±0.00^{a}	12.36±4.77^{c}	9.98±2.65^{c}	0.00±0.00^{a}	0.00±0.00^{a}	0.00±0.00^{a}	7.20±0.84^{b}
Nonan-1-ol	AL8	38.01	1660	0.00±0.00^{a}	0.00±0.00^{a}	0.00±0.00^{a}	0.00±0.00^{a}	0.00±0.00^{a}	0.00±0.00^{a}	0.00±0.00^{a}	1.54±0.60^{b}	0.00±0.00^{a}	0.00±0.00^{a}	0.00±0.00^{a}	2.30±0.85^{c}
(2E)-3,7-Dimethylocta-2,6-dien-1-ol	AL9	47.96	1847	3.87±0.79^{a}	3.10±1.36^{a}	14.61±3.40^{c,d,e}	8.01±5.63^{a,b,c}	3.17±1.20^{a}	5.09±0.51^{a,b}	19.47±10.70^{e}	11.80±4.69^{b,c,d}	4.31±3.05^{a,b}	8.07±0.93^{a,b,c}	18.21±5.24^{d,e}	17.64±2.59^{d,e}
Aldehydes															
(2E)-Hept-2-enal	AH1	18.12	1323	0.00±0.00^{a}	0.00±0.00^{a}	0.00±0.00^{a}	2.09±2.66^{b}	0.00±0.00^{a}	0.00±0.00^{a}	5.76±1.40^{c}	2.75±0.68^{b}	0.00±0.00^{a}	0.00±0.00^{a}	0.00±0.00^{a}	4.64±2.67^{c}
(2E,4E)-Hepta-2,4-dienal	AH2	28.01	1495	0.00±0.00^{a}	0.00±0.00^{a}	2.42±0.66^{b}	0.00±0.00^{a}	0.00±0.00^{a}	0.00±0.00^{a}	7.18±0.73^{c}	0.00±0.00^{a}	0.00±0.00^{a}	0.00±0.00^{a}	0.00±0.00^{a}	0.00±0.00^{a}
(2E,6Z)-Nona-2,6-dienal	AH3	33.29	1584	0.00±0.00^{a}	0.00±0.00^{a}	0.00±0.00^{a}	0.00±0.00^{a}	0.00±0.00^{a}	0.00±0.00^{a}	2.64±1.25^{b}	0.00±0.00^{a}	0.00±0.00^{a}	0.00±0.00^{a}	0.00±0.00^{a}	0.00±0.00^{a}
(2Z)-3,7-Dimethylocta-2,6-dienal	AH4	38.51	1680	4.17±0.91^{a,b}	20.75±11.40^{d}	10.42±2.01^{b,c}	2.39±0.91^{a,b}	5.94±2.74^{a,b}	1.70±0.11^{a,b}	15.67±12.18^{c,d}	0.90±0.42^{a,b}	8.80±4.83^{a,b,c}	2.23±0.59^{a,b}	2.66±0.36^{a,b}	0.00±0.00^{a}
(E)-3,7-Dimethylocta-2,6-dienal	AH5	41.36	1732	6.31±1.03^{a}	29.87±13.17^{c}	25.23±7.57^{b,c}	4.07±1.12^{a}	11.99±7.22^{a,b}	4.12±0.55^{a}	29.02±21.27^{c}	0.00±0.00^{a}	12.64±5.59^{a,b}	7.14±3.60^{a}	5.49±0.12^{a}	0.00±0.00^{a}
Isothiocyanate															
2-Isothiocyanatobutane	IT1	15.01	1287	11.72±3.63^{c,d}	4.17±2.22^{a,b}	9.66±2.02^{c}	2.62±1.52^{a}	9.28±5.67^{c}	4.61±3.13^{a,b}	7.80±2.85^{b,c}	3.62±0.96^{a,b}	15.25±2.48^{d}	3.20±1.00^{a,b}	4.20±0.43^{a,b}	2.99±0.68^{a,b}
4-Isothiocyanatobut-1-ene	IT2	25.99	1435	63.42±23.50^{b,c}	54.85±18.04^{b,c}	8.61±5.65^{a}	1.78±1.04^{a}	37.13±18.16^{a,b}	98.83±25.69^{d}	3.67±1.93^{a}	0.00±0.00^{a}	136.69±34.85^{e}	105.24±19.51^{d,e}	73.01±51.19^{c,d}	6.47±3.38^{a}
1-Isothiocyanatoheptane	IT3	36.46	1666	2.59±0.61^{c,d}	1.54±0.66^{b}	0.00±0.00^{a}	0.00±0.00^{a}	2.21±0.98^{b,c}	2.08±0.77^{b,c}	0.00±0.00^{a}	0.00±0.00^{a}	1.96±0.83^{b,c}	2.01±0.59^{b,c}	3.31±0.96^{d}	0.00±0.00^{a}
2-(Isothiocyanato)ethylbenzene	IT4	66.38	2234	35.49±9.59^{a,b}	40.85±6.34^{a,b}	35.48±9.17^{a,b}	23.33±8.60^{a,b}	53.28±48.81^{b,c}	76.08±34.75^{c}	28.81±7.82^{a,b}	10.91±2.95^{a}	88.21±21.47^{c}	81.30±7.85^{c}	84.28±3.84^{c}	38.89±4.53^{a,b}

Volatile compounds (ppm Methyl cinnamate equivalents)	Abbreviation	RT	RI	Non-starter				*Leuconostoc lactis* DMLL10				*Leuconostoc mesenteroides* KACC 12312^{T}			
				1 day	5 day	10 day	20 day	1 day	5 day	10 day	20 day	1 day	5 day	10 day	20 day
Ketones															
6-Methylhept-5-en-2-one	K1	19.12	1338	1.81±0.44^{a,b,c}	4.90±2.31^{d,e}	6.01±1.89^{e}	0.75±0.97^{a}	3.65±1.49^{c,d}	1.51±0.53^{a,b}	3.47±1.25^{b,c,d}	1.34±0.24^{a}	2.35±0.21^{a,b,c}	1.14±0.06^{a}	2.51±0.66^{a,b,c}	1.88±0.90^{a,b,c}
Undecan-2-one	K2	34.16	1598	1.13±0.36^{a,b}	2.87±1.21^{c}	5.08±1.55^{d}	0.00±0.00^{a}	2.18±0.79^{b,c}	1.04±0.15^{a,b}	0.00±0.00^{a}	0.00±0.00^{a}	1.77±0.06^{b}	1.02±0.08^{a,b}	0.00±0.00^{a}	0.00±0.00^{a}
Nitrile compounds															
Pent-4-enenitrile	N1	19.53	858	18.69±4.51^{b,c,d}	2.99±0.63^{a}	19.90±2.61^{b,c,d}	10.64±2.00^{a,b,c}	14.70±7.16^{a,b,c,d}	6.36±3.35^{a,b}	28.57±4.45^{d}	13.94±1.58^{a,b,c,d}	17.09±5.92^{a,b,c,d}	7.64±4.16^{a,b}	27.61±15.49^{d}	23.50±22.58^{c,d}
5-(Methylthio)pentanenitrile	N2	51.28	1851	1.16±0.28^{b}	0.00±0.00^{a}	0.00±0.00^{a}	0.00±0.00^{a}	0.00±0.00^{a}	0.00±0.00^{a}	0.00±0.00^{a}	0.00±0.00^{a}	0.00±0.00^{a}	0.00±0.00^{a}	0.00±0.00^{a}	0.00±0.00^{a}
3-Phenylpropanenitrile	N3	56.50	2041	30.35±7.72^{c,d}	5.85±1.87^{a}	15.46±2.24^{a,b}	11.09±6.04^{a,b}	22.99±12.50^{b,c,d}	9.04±0.73^{a,b}	11.64±10.11^{a,b}	13.38±3.54^{a,b}	32.21±16.72^{d}	12.14±0.21^{a,b}	18.05±5.34^{a,b,c}	10.32±1.46^{a,b}
6-(Methylthio)hexanenitrile	N4	57.40	2089	7.10±3.58^{d}	0.78±0.35^{a,b}	1.87±0.05^{a,b}	0.00±0.00^{a}	3.45±2.12^{b,c}	1.65±0.55^{a,b}	2.75±1.55^{a,b,c}	1.77±0.71^{a,b}	5.35±2.63^{c,d}	1.61±0.19^{a,b}	2.55±0.06^{a,b}	0.00±0.00^{a}
Sulfur compounds															
ethene sulfide	S1	2.77	908	44.91±42.00^{b,c}	0.00±0.00^{a}	0.00±0.00^{a}	0.00±0.00^{a}	8.13±1.99^{a,b}	4.89±0.94^{a}	13.55±4.94^{a,b}	0.00±0.00^{a}	80.76±60.44^{c}	0.00±0.00^{a}	0.00±0.00^{a}	0.00±0.00^{a}
1-Propenyl 1-propenyl sulfide	S2	9.39	1148	42.74±14.23^{a,b,c}	19.30±7.70^{a,b}	41.68±55.87^{a,b,c}	45.86±16.38^{a,b,c}	12.97±5.73^{a}	17.90±0.94^{a,b}	59.45±23.02^{c}	51.74±12.69^{b,c}	53.83±4.10^{b,c}	17.37±4.87^{a,b}	32.02±10.00^{a,b,c}	32.54±8.05^{a,b,c}
Methyl propyl disulfide	S3	13.01	1239	24.69±3.98^{a}	20.57±14.76^{a}	74.04±16.42^{c}	29.16±15.29^{a,b}	30.38±19.71^{a,b}	22.51±4.71^{a}	45.21±15.12^{b}	32.21±7.50^{a,b}	23.00±4.73^{a}	14.91±2.35^{a}	23.94±4.90^{a}	18.39±4.37^{a}
Methyl thiocyanate	S4	15.41	1276	0.00±0.00^{a}	1.88±0.43^{a,b,c}	6.74±1.26^{g}	4.00±0.71^{c,d,e}	0.00±0.00^{a}	2.09±1.31^{a,b,c,d}	6.38±1.15^{f,g}	3.52±0.30^{b,c,d}	0.00±0.00^{a}	1.71±0.02^{a,b}	4.24±2.23^{d,e,f}	5.88±3.22^{e,f,g}
Methyl 2-propenyl disulfide	S5	15.69	1281	154.47±16.61^{a,b}	148.51±71.29^{a,b}	385.80±95.36^{c}	159.73±73.32^{a,b}	131.28±86.45^{a,b}	150.45±19.55^{a,b}	327.17±108.20^{c}	164.93±37.95^{a,b}	227.00±33.24^{b}	120.35±13.87^{a,b}	200.30±63.88^{a,b}	100.00±24.63^{a}
Methanethio thio methanethiol	S6	20.88	1377	0.00±0.00^{a}	0.00±0.00^{a}	0.00±0.00^{a}	1.79±0.66^{a,b}	10.75±16.49^{b}	1.88±0.44^{a,b}	4.01±1.19^{a,b}	3.10±0.64^{a,b}	3.16±1.22^{a,b}	1.26±0.10^{a}	1.83±0.38^{a,b}	2.68±1.15^{a,b}
Dipropyl disulfide	S7	21.07	1378	79.80±55.68^{a,b}	48.19±34.30^{a}	248.90±65.99^{d}	149.38±71.76^{b,c}	109.65±62.71^{a,b,c}	78.45±23.24^{a,b}	178.77±45.77^{c,d}	113.89±48.94^{a,b,c}	103.25±48.22^{a,b,c}	58.86±8.78^{a}	77.75±10.93^{a,b}	73.78±9.59^{a,b}
(E)-1-(Prop-1-en-1-yl)-2-propyldisulfane	S8	22.98	1118	7.05±2.06^{c,d}	2.65±1.79^{a}	10.67±2.18^{e}	6.46±1.90^{b,c}	5.07±0.82^{a,b,c}	2.86±1.23^{a}	9.86±2.43^{d,e}	3.91±1.44^{a,b,c}	5.11±3.71^{a,b,c}	4.02±1.75^{a,b,c}	3.09±0.64^{a,b}	3.33±1.21^{a,b}
1-Allyl-2-isopropyldisulfane	S9	24.11	1076	157.96±46.45^{a,b,c}	84.92±39.16^{a}	413.20±121.98^{e}	280.60±132.44^{c,d}	102.49±72.00^{a,b}	117.81±29.55^{a,b}	380.80±121.76^{d,e}	224.35±87.95^{b,c}	104.41±36.31^{a,b}	114.79±10.39^{a,b}	146.78±36.81^{a,b}	106.42±15.17^{a,b}
1-(Prop-2-en-1-yl)disulfanylprop-2-ene	S10	27.32	1475	648.14±87.32^{a,b}	555.24±176.67^{a,b}	1316.73±388.27^{c}	1348.46±447.87^{c}	385.80±223.29^{a}	712.16±148.10^{a,b}	1277.51±518.75^{c}	848.51±265.89^{a,b,c}	950.38±156.02^{b,c}	668.29±64.76^{a,b}	965.92±487.65^{b,c}	665.86±162.63^{a,b}
(E)-1-Allyl-2-(prop-1-en-1-yl)disulfane	S11	27.50	1103	67.57±15.90^{a,b,c}	46.43±22.13^{a,b}	120.36±44.90^{c,d}	143.23±48.42^{d}	42.50±30.66^{a,b}	55.82±8.64^{a,b}	142.82±59.62^{d}	68.43±33.39^{a,b,c}	93.98±32.89^{b,c,d}	62.35±8.20^{a,b}	69.04±26.32^{a,b,c}	31.19±1.85^{a}
2-Ethyl-1,3-dithiane	S12	28.95	1130	2.45±0.20^{a,b}	1.46±0.73^{a}	5.79±1.01^{d}	4.64±0.44^{c,d}	1.21±0.25^{a}	1.81±0.23^{a,b}	8.56±2.95^{e}	5.07±1.94^{d}	2.54±0.43^{a,b}	2.33±0.37^{a,b}	3.79±1.16^{b,c,d}	2.90±0.46^{a,b,c}
3H-1,2-dithiole	S13	29.31	1510	37.01±10.24^{b}	22.81±8.90^{a,b}	43.34±13.53^{b,c}	63.22±22.27^{c}	21.41±9.29^{a,b}	28.36±3.96^{a,b}	63.35±28.33^{c}	28.00±14.66^{a,b}	47.62±22.43^{b,c}	25.45±3.96^{a,b}	24.59±9.14^{a,b}	8.52±1.69^{a}
4-Ethyl-5-methyl-1,3-thiazole	S14	29.86	1412	1.30±0.51^{b}	0.00±0.00^{a}	0.00±0.00^{a}	0.00±0.00^{a}	0.00±0.00^{a}	0.00±0.00^{a}	0.00±0.00^{a}	0.00±0.00^{a}	0.00±0.00^{a}	0.00±0.00^{a}	0.00±0.00^{a}	0.00±0.00^{a}
Methyl 2-propenyl trisulfide	S15	33.01	1593	0.00±0.00^{a}	0.00±0.00^{a}	0.00±0.00^{a}	94.69±19.11^{d}	49.85±43.35^{c}	41.01±7.53^{b,c}	0.00±0.00^{a}	20.13±5.71^{a,b}	0.00±0.00^{a}	43.32±9.32^{b,c}	0.00±0.00^{a}	13.53±0.34^{a}
Hexahydropyrrolizine-3-thione	S16	37.23	1222	13.15±4.21^{b}	0.00±0.00^{a}	0.00±0.00^{a}	0.00±0.00^{a}	12.38±6.36^{b}	13.43±4.75^{b}	0.00±0.00^{a}	0.00±0.00^{a}	15.80±8.43^{b}	0.00±0.00^{a}	0.00±0.00^{a}	0.00±0.00^{a}
Dipropyl trisulfide	S17	37.29	1703	0.00±0.00^{a}	0.00±0.00^{a}	16.27±4.21^{d}	10.32±3.94^{b,c}	0.00±0.00^{a}	0.00±0.00^{a}	15.06±5.16^{d}	6.26±3.15^{b}	0.00±0.00^{a}	0.00±0.00^{a}	13.30±0.66^{c,d}	8.79±2.12^{b}

Volatile compounds (ppm Methyl cinnamate equivalents)	Abbreviation	RT	RI	Non-starter				*Leuconostoc lactis* DMLL10				*Leuconostoc mesenteroides* KACC 12312^{T}			
				1 day	5 day	10 day	20 day	1 day	5 day	10 day	20 day	1 day	5 day	10 day	20 day
3-Vinyl-1,2-dithiacyclohex-4-ene	S18	40.72	1750	47.43±6.61^{a}	0.00±0.00^{a}	49.24±9.41^{a}	72.84±37.74^{a,b}	15.26±0.84^{a}	34.73±2.75^{a}	135.48±149.52^{b}	20.72±15.86^{a}	55.83±18.42^{a}	27.28±7.24^{a}	35.67±16.59^{a}	15.05±2.45^{a}
Di-2-propenyl trisulfide	S19	43.93	1805	158.82±27.37^{a,b,c}	101.25±32.92^{a,b}	243.06±79.46^{c,d,e}	342.54±54.62^{e}	97.93±52.45^{a}	154.53±36.48^{a,b,c}	282.05±117.79^{d,e}	125.24±50.22^{a,b}	217.60±96.64^{b,c,d}	142.19±16.89^{a,b,c}	161.59±97.10^{a,b,c}	72.13±13.90^{a}
4-Vinyl-4H-1,3-dithiin	S20	46.53	1857	42.65±4.01^{a,b}	34.27±9.77^{a}	96.09±31.12^{c}	72.08±39.57^{b,c}	22.03±10.06^{a}	44.89±1.97^{a,b}	101.06±38.47^{c}	41.27±13.18^{a,b}	50.95±22.57^{a,b}	36.24±6.73^{a,b}	54.72±25.00^{a,b}	30.78±0.99^{a}
Methyl 1-[(1-propenyl)thio]propyl disulfide	S21	48.17	1407	0.00±0.00^{a}	0.38±0.13^{a,b}	1.32±0.29^{a,b}	0.00±0.00^{a}	0.00±0.00^{a}	0.00±0.00^{a}	1.91±1.04^{b}	1.06±0.39^{a,b}	0.00±0.00^{a}	0.00±0.00^{a}	4.43±4.06^{c}	0.00±0.00^{a}
Methyl 1-(methylthio)propyl disulfide	S22	50.21	1235	0.00±0.00^{a}	0.00±0.00^{a}	2.98±0.86^{c}	1.35±0.47^{b}	0.00±0.00^{a}	0.00±0.00^{a}	4.12±1.24^{d}	1.75±1.10^{b}	0.00±0.00^{a}	1.73±0.25^{b}	0.00±0.00^{a}	0.00±0.00^{a}
1,3-Dithiole-2-thione	S23	78.60	1172	0.00±0.00^{a}	0.73±0.23^{b}	1.58±0.31^{c}	0.00±0.00^{a}	0.00±0.00^{a}	0.00±0.00^{a}	1.72±0.65^{c}	0.00±0.00^{a}	1.62±0.25^{c}	0.00±0.00^{a}	0.00±0.00^{a}	0.00±0.00^{a}
Terpenes															
2,2-Dimethyl-3-methylenebicyclo[2.2.1]heptane	T1	5.90	1071	22.45±11.69^{a}	46.77±31.77^{a,b,c}	88.00±52.59^{c}	12.16±3.72^{a}	39.65±37.49^{a,b}	18.62±7.34^{a}	71.30±19.04^{b,c}	23.65±3.03^{a}	51.11±32.25^{a,b,c}	15.15±3.05^{a}	15.60±9.93^{a}	43.59±13.68^{a,b,c}
(1S,5S)-2-Methyl-5-((R)-6-methylhept-5-en-2-yl)bicyclo[3.1.0]hex-2-ene	T2	31.59	1389	0.84±0.13^{a,b}	2.52±1.92^{b}	5.47±3.25^{c}	0.00±0.00^{a}	1.60±1.30^{a,b}	0.00±0.00^{a}	2.61±0.33^{b}	0.00±0.00^{a}	1.19±0.25^{a,b}	1.15±0.52^{a,b}	0.00±0.00^{a}	0.00±0.00^{a}
(6Z,10Z)-3,7,11-Trimethyldodeca-1,6,10-triene	T3	38.12	1662	0.00±0.00^{a}	3.39±3.88^{b}	3.45±3.23^{b}	0.00±0.00^{a}	0.00±0.00^{a}	0.00±0.00^{a}	0.00±0.00^{a}	0.00±0.00^{a}	0.00±0.00^{a}	0.00±0.00^{a}	0.00±0.00^{a}	0.00±0.00^{a}
(E)-1-Methyl-4-(6-methylhepta-1,5-dien-2-yl)cyclohexene	T4	40.89	1727	8.20±2.83^{a}	35.84±18.29^{a}	76.40±32.18^{b}	6.03±0.23^{a}	23.27±20.53^{a}	11.81±4.61^{a}	74.05±38.23^{b}	21.41±11.39^{a}	15.87±2.97^{a}	21.57±6.80^{a}	17.68±4.19^{a}	22.45±5.30^{a}
(6E,10E)-3,7,11-Trimethyldodeca-1,6,10-triene	T5	42.56	1746	7.26±0.39^{a}	29.02±15.05^{a,b}	44.23±23.52^{b,c}	3.56±0.92^{a}	9.44±11.85^{a}	10.36±3.26^{a}	67.08±39.99^{c}	15.63±8.29^{a}	10.23±4.75^{a}	15.76±4.22^{a}	10.59±3.46^{a}	15.70±4.19^{a}

All values were determined three times independently and the mean values of the replicates are presented. Different letter in each row indicates significant difference at $p < 0.05$ using Duncan's multiple range test.

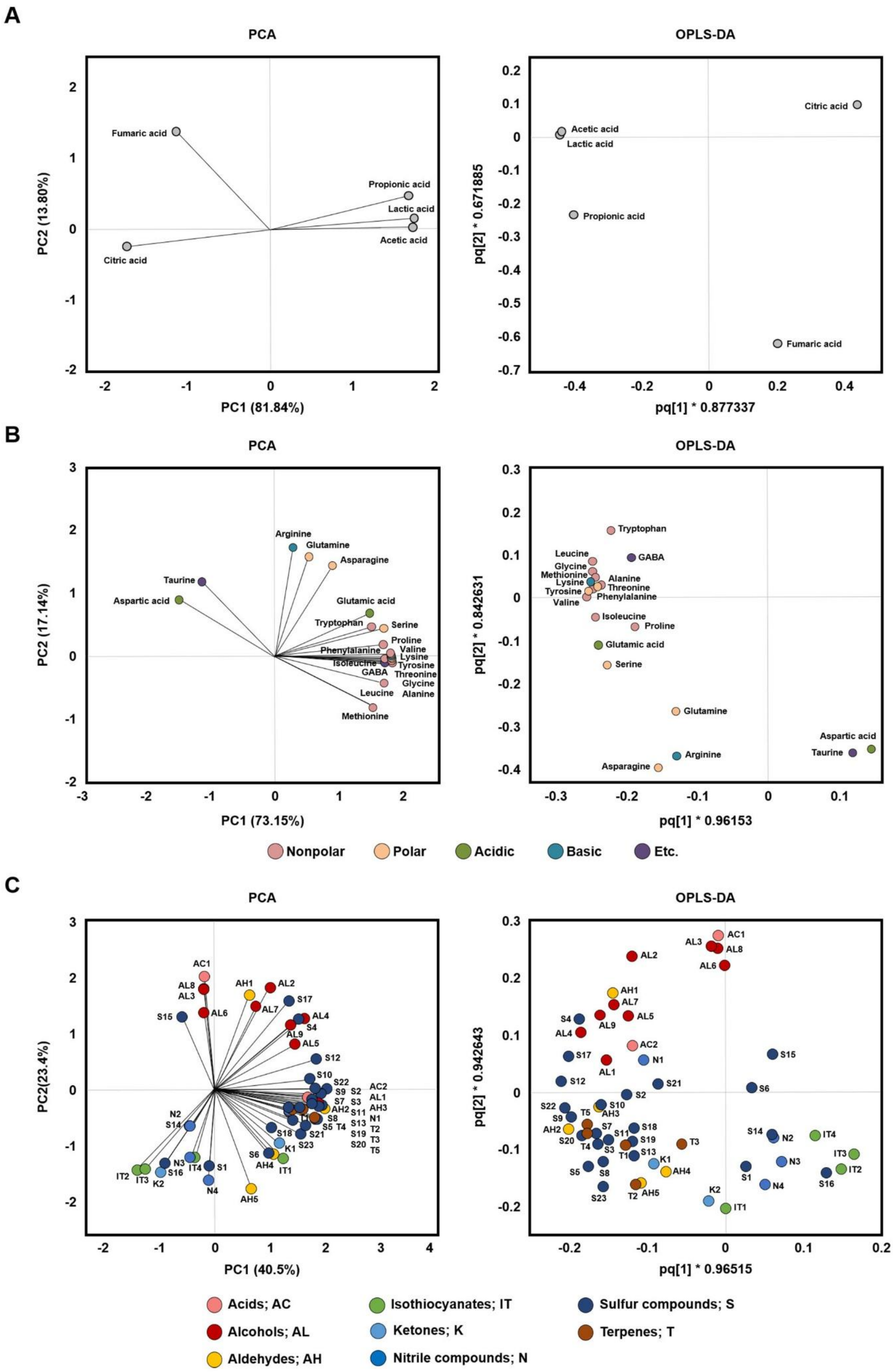
A
PCA
OPLS-DA
PC1 (81.84%)
PC2 (13.80%)
Fumaric acid
Propionic acid
Lactic acid
Acetic acid
Citric acid
pq[1] * 0.877337
pq[2] * 0.671885
B
PCA
OPLS-DA
PC1 (73.15%)
PC2 (17.14%)
pq[1] * 0.96153
pq[2] * 0.842631
Arginine
Glutamine
Asparagine
Taurine
Aspartic acid
Glutamic acid
Tryptophan
Serine
Proline
Phenylalanine
Valine
Lysine
Isoleucine
Tyrosine
GABA
Threonine
Glycine
Leucine
Alanine
Methionine
Nonpolar
Polar
Acidic
Basic
Etc.
C
PCA
OPLS-DA
PC1 (40.5%)
PC2(23.4%)
pq[1] * 0.96515
pq[2] * 0.942643
Acids; AC
Alcohols; AL
Aldehydes; AH
Isothiocyanates; IT
Ketones; K
Nitrile compounds; N
Sulfur compounds; S
Terpenes; T

Fig. S1. Principal component analysis (PCA) and orthogonal partial least squares discriminant analysis (OPLS-DA) loading plots based on organic acid production (A), amino acid production (B) and volatile compound production (C). All analyses were performed using triplicate data.
